# High-Resolution 3D Bioprinted Hydrogel Scaffolds Enable Sustained Intraperitoneal Cell Delivery

**DOI:** 10.3390/molecules31111958

**Published:** 2026-06-04

**Authors:** Yu Zhang, Lauren E. Carlberg, Cali N. Colliver, Alain Valdivia, Morrent Thang, Caroline A. Stockwell, Jillian L. Perry, Shawn D. Hingtgen

**Affiliations:** 1 Division of Pharmacoengineering and Molecular Pharmaceutics, UNC Eshelman School of Pharmacy, The University of North Carolina at Chapel Hill, Chapel Hill, NC 27599, USA; zycpu@email.unc.edu (Y.Z.); lekass@email.unc.edu (L.E.C.); alain07@email.unc.edu (A.V.); cstockw@unc.edu (C.A.S.); 2 Department of Chemistry, The University of North Carolina at Chapel Hill, Chapel Hill, NC 27599, USA; 3 Center for Nanotechnology in Drug Delivery, Eshelman School of Pharmacy, The University of North Carolina at Chapel Hill, Chapel Hill, NC 27599, USA; 4 Lineberger Comprehensive Cancer Center, The University of North Carolina at Chapel Hill, Chapel Hill, NC 27599, USA

**Keywords:** Continuous Liquid Interface Printing (CLIP), 3D bioprinting, hydrogel scaffolds, cell delivery, intraperitoneal implant

## Abstract

Intraperitoneal (I.P.) delivery of cell-based therapeutics represents a promising strategy for treating regional peritoneal diseases; however, rapid cellular clearance severely limits therapeutic durability. A critical unmet need is the development of implantable biomaterial platforms that can both mechanically integrate within the dynamic I.P. cavity and sustain viable cell persistence in vivo. Here, we establish a Continuous Liquid Interface Production (CLIP)-based 3D bioprinting strategy to engineer transplantable, cell-laden hydrogel scaffolds optimized for I.P. implantation. Through systematic bioresin design, we identify a GelMA-PEGDA formulation that achieves a balance between high-resolution printability, tissue-matched mechanical characteristics (Young’s modulus 10–15 kPa), and controlled biodegradation (~75% mass loss over 14 days). The resulting constructs support sustained cell viability and proliferation for over 30 days in vitro. Importantly, in an animal study conducted in 6–8 weeks of female nude mice, in vivo I.P. implantation demonstrates a ~10-fold extension in cellular persistence compared to direct cell injection, prolonging the time to 50% signal decay from ~3 days to ~30 days, with detectable cell retention approaching two months in select animals. The platform further accommodates multiple clinically relevant cell types, including human mesenchymal stem cells and neural stem cells, highlighting its translational versatility. Collectively, this work defines key material and architectural parameters required for I.P. implantable cell therapeutics and establishes CLIP-based bioprinting as a scalable strategy for regional delivery of living therapeutics.

## 1. Introduction

Cell-based therapies are emerging as versatile treatment modalities across a wide range of conditions [1], including diseases localized to the peritoneal cavity. As living medicines, therapeutic cells can persist, adapt, and exert sustained biological activity in vivo. However, effective delivery remains a major constraint. Standard intravenous infusion is often ineffective for peritoneal disease, as cells must survive vascular shear, evade immune clearance, and extravasate across multiple tissue barriers, which all lead to limited localization at the target site [2]. Direct intraperitoneal (I.P.) administration enables high local cell deposition [3,4]. Consequently, a growing body of clinical and preclinical studies has explored the I.P. delivery of diverse cell types, from immune effectors to regenerative stem cells, for a spectrum of peritoneal diseases. Table 1 provides a summary of these key applications, highlighting the breadth of this therapeutic modality.

Despite its promise, the clinical translation of I.P. cell therapy faces a major bottleneck. The I.P. cavity presents a uniquely hostile environment. It is a large, fluid-filled, immunologically active, and anatomically complex space that promotes uneven cell dispersion and rapid clearance [25,26,27,28,29,30,31,32]. Regardless of the cell type, free cells administered into the peritoneal cavity are subject to immediate and substantial loss. For instance, studies on mesenchymal stem cells (MSCs) have shown that living MSCs diminished significantly by day 3 and became undetectable by day 7 in the I.P. cavity [33], a process driven by apoptosis and clearance by resident macrophages [34]. This phenomenon is not unique to stem cells. The transplantation of unmodified pancreatic islets into the peritoneal cavity has consistently resulted in poor engraftment and function, with over 50% of islets failing to engraft in the early post-transplant period [35,36,37]. Similarly, free hepatocytes transplanted into the peritoneal cavity for liver failure models show a rapid loss of viability, with over 70% of cells rapidly cleared [38,39], necessitating the use of encapsulation technologies to improve survival [15]. This rapid clearance is mediated by several factors, including phagocytosis by a large population of resident peritoneal macrophages, mechanical clearance via lymphatic drainage, and adhesion to peritoneal surfaces, particularly the omentum, which serves as a primary site for immune surveillance and foreign material clearance [27]. Therefore, a critical unmet need exists for advanced delivery platforms that can protect therapeutic cells from these clearance mechanisms and prolong their residence time and therapeutic activity within the peritoneal cavity.

To overcome the rapid cell clearance in the I.P. cavity, drug delivery systems (DDS) such as hydrogels have emerged as a compelling strategy [3,40,41]. Hydrogels are water-swollen, crosslinked polymer networks that closely mimic the hydrated, three-dimensional architecture of the native extracellular matrix (ECM), providing encapsulated cells with a supportive microenvironment that promotes survival, attachment, and function [42,43]. When implanted intraperitoneally, hydrogel scaffolds serve as a physical barrier that shields therapeutic cells from the rapid clearance and mechanical forces of the peritoneal cavity. Simultaneously, the hydrogel scaffolds function as localized living drug depots, maintaining high therapeutic concentrations within the I.P. cavity while limiting systemic off-target toxicity [44]. This approach has shown considerable promise. Alginate-based capsules have been used to encapsulate islet cells for I.P. transplantation in diabetes [45], and more recently, engineered “cytokine factories”, alginate capsules containing IL-2-producing cells, have demonstrated the ability to eradicate I.P. tumors in preclinical models [6]. However, these existing I.P. hydrogel delivery systems predominantly rely on simple fabrication methods such as ionic crosslinking [6,45] or in situ gelling after injection [46], which offer limited control, balancing scaffold architecture, mechanical properties, as well as homogenous crosslinking and drug loading [47]. The ideal hydrogel scaffolds for the dynamic I.P. environment must satisfy a more comprehensive set of design criteria: they must be biocompatible, have defined architecture, possess tunable degradation kinetics, and exhibit physiologically relevant mechanical properties to withstand peritoneal forces without inducing a significant foreign body response (FBR).

Hydrogel degradation is essential for post-implantation performance, as matrix remodeling enables therapeutic cells and proteins to migrate out of the scaffold and engage with the surrounding environment [42,48]. Controlled degradation also decreases crosslink density and enhances nutrient, oxygen, and waste transport, thereby sustaining long-term cell viability and therapeutic activity [49]. Swelling is another key parameter influencing the scaffold’s mechanical stability and nutrient transport, factors that directly impact cell viability and therapeutic efficacy. Excessive swelling may compromise implant localization or irritate the peritoneal environment. Furthermore, biomaterial stiffness is a critical regulator of host response because it dictates how macrophages sense and respond to the implant through mechanotransduction pathways, thereby shaping inflammatory activation and fibrosis formation [50], also referred to as FBR. When stiffness is substantially higher than the surrounding tissue, the mechanical mismatch amplifies macrophage pro-inflammatory signaling, accelerates foreign body giant cell formation, and promotes dense fibrotic encapsulation [51], ultimately impairing nutrient diffusion, cell release, and overall therapeutic efficacy. In contrast, materials within the native tissue stiffness range evoke a more balanced macrophage response and reduced fibrosis [50]. Therefore, for I.P. implantation, hydrogels should exhibit mechanical properties comparable to native peritoneal tissues, which typically have a modulus ranging from 1 to 15 kPa [52,53]. Although very soft hydrogels (<1 kPa) minimize FBR, they often lack the mechanical integrity required for surgical handling and I.P. placement. Therefore, scaffold architecture and material chemistry must be precisely tuned to the unique demands of the I.P. microenvironment, cell bioactivity, and surgical implantation.

Fabricating scaffolds that meet these multifaceted requirements simultaneously presents a considerable biofabrication challenge. Conventional techniques like solvent casting offer poor architectural control [54], and while established 3D bioprinting methods like extrusion-based printing offer better control, they often impart high shear stress on cells and are limited in resolution and fabrication speed [55,56]. Continuous Liquid Interface Production (CLIP), a vat polymerization-based 3D printing technology, offers a powerful alternative to these challenges [57] (Schematic Abstract, Top). CLIP builds structures through continuous photopolymerization at a liquid-solid interface using an oxygen-permeable window, which creates a thin oxygen rich “dead zone” where polymerization is inhibited. This allows rapid printing of complex three-dimensional architectures. This approach allows precise control over scaffold architecture, material composition, and crosslink density, while maintaining high cell encapsulation and viability due to the absence of mechanical shear [58]. While our group and others have previously utilized CLIP to bioprint scaffolds for implantation into confined, static environments like post-surgical brain cavities [59], its adaptation for the fundamentally different demands of the open and dynamic I.P. space remains unexplored.

In this work, we aimed to develop and systematically characterize a CLIP-based 3D bioprinting platform specifically tailored for fabricating cell-laden hydrogel scaffolds for extended intraperitoneal delivery. We hypothesized that by optimizing the bioresin formulation and printing parameters, we could produce scaffolds with the precise architectural and mechanical properties required to prolong cell persistence in the I.P. cavity. To achieve this, we first developed a GelMA-PEGDA bioresin and characterized its printability, degradation, swelling, and Young’s modulus. With a model human fibroblast cell line NHF1^GFP-FL^, we then evaluated the platform’s ability to support long-term cell viability and proliferation in vitro before demonstrating markedly improved cell persistence in vivo following I.P. implantation in healthy nude mice. Nude mice are used as a recipient for human cells to avoid rejection of foreign cell. Finally, we confirmed the platform’s versatility by successfully bioprinting multiple clinically relevant cell types. This study establishes a robust and scalable biofabrication strategy for engineering the next generation of implantable cell delivery systems for regional therapies.

## 2. Results

### 2.1. Development of a Biocompatible Bioprinting Resin Formulation

To explore the potential of using CLIP for 3D bioprinting of living cells, we first screened resin formulations to identify a composition that maintained high viability (greater than 80%) of encapsulated cells. For these investigations, we utilized NHF1^GFP-FL^ cells and monitored viability via bioluminescence (BLI) signals. We characterized the direct cytotoxicity of key bioprinting variables, including exposure to monomers (GelMA and PEGDA), the photoinitiator (LAP), the photoabsorber (Ecamsule), and UV light.

We selected several biocompatible monomers, including GelMA and two molecular weights of PEGDA (6K and 10K), all of which have been largely employed in UV-based additive manufacturing systems for drug delivery, tissue engineering, and 3D bioprinting [60,61,62,63]. GelMA was selected for its biocompatibility, biodegradability, and provision of an extracellular matrix-mimetic environment containing cell adhesion motifs essential for cell attachment and expansion. High molecular weight PEGDA was included to enhance the physical characteristics and printability of the resin. LAP was selected as a common, water-soluble photoinitiator [64]. Ecamsule, a water-soluble, FDA-approved UV-filter used in commercial sunscreens [65], was for its potential to absorb excessive scattered UV light during printing to improve resolution; notably, this represents the first reported use of Ecamsule in a 3D bioprinting system.

To identify cytocompatible concentrations, we prepared material solutions across a concentration gradient and incubated them with NHF1^GFP-FL^ cells for an hour, establishing a threshold of 80% cell viability. To investigate the combined impact of materials and UV light, we also exposed the cell-material mixtures to 100% intensity 365 nm UV light for 60 s.

**Figure 1:**
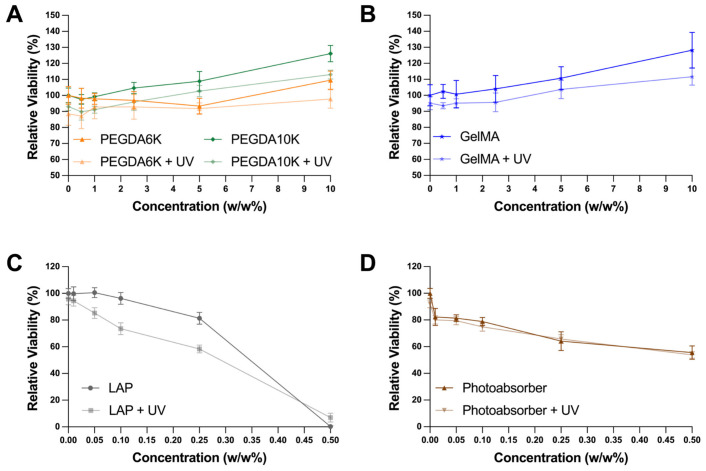
Screening cytocompatibility of resin components for 3DBP. Summary BLI images and graphs showing that adjustments in PEGDAs (**A**), GelMA (**B**), photoinitiator (**C**), and photoabsorber (**D**) retain high NHF1^GFP-FL^ viability.

We observed similar levels of viability for cells incubated with low concentrations of PEGDA6K, PEGDA10K, and GelMA compared to the non-treated control (0 *w*/*w*%), with viability increasing at higher monomer concentrations (Figure 1A,B). Cells incubated with LAP exhibited a dose-dependent decrease in viability (Figure 1C); concentrations below 0.1 *w*/*w*% maintained ~100% viability, whereas viability dropped significantly to near 0% at 0.5 *w*/*w*%, demonstrating strong cytotoxicity at higher concentrations, consistent with previous reports [66]. Cells incubated with Ecamsule showed a stepwise decrease in viability from 100% to 55% as the concentration increased from 0 to 0.5 *w*/*w*% (Figure 1D), indicating dose-dependent cytotoxicity.

We next explored the combined impact of these materials with UV exposure. For cells incubated with the monomers (GelMA, PEGDA6K, and PEGDA10K), UV exposure resulted in only a ~10% decrease in viability across all tested concentrations, indicating that UV light caused a minor level of cell death without a synergistic cytotoxic effect with the materials. Following UV exposure, cells incubated with LAP showed a significant drop in viability compared to non-UV-exposed groups; this was expected, as UV light decomposes LAP into cytotoxic free radicals. Interestingly, for cells incubated with Ecamsule, we observed no significant difference in viability between UV-exposed and non-exposed groups across all tested concentrations. This suggests that even at low concentrations, Ecamsule effectively absorbs UV light, thereby limiting cellular UV exposure and preventing additional cell death.

It is important to note that high-intensity UV exposure used and the 1 h culture of cells within the resin applied in these screening studies represent more stringent conditions than actual bioprinting. During CLIP bioprinting, cells are in contact with the resin for only a few minutes, and the CLIP printer utilizes a 385 nm light source at a significantly lower intensity than the 100% intensity UV source used for screening. Furthermore, during printing, cells within the resin are exposed to this lower-intensity light for a maximum of approximately 5 min depending on part architecture. Therefore, we anticipate reduced cytotoxicity under actual bioprinting conditions. Based on these findings, we selected 0.25 *w*/*w*% LAP and 0.5 *w*/*w*% Ecamsule for initial scaffold development.

### 2.2. Evaluation of Printability and Cell Viability with Various Resin Formulations

**Figure 2:**
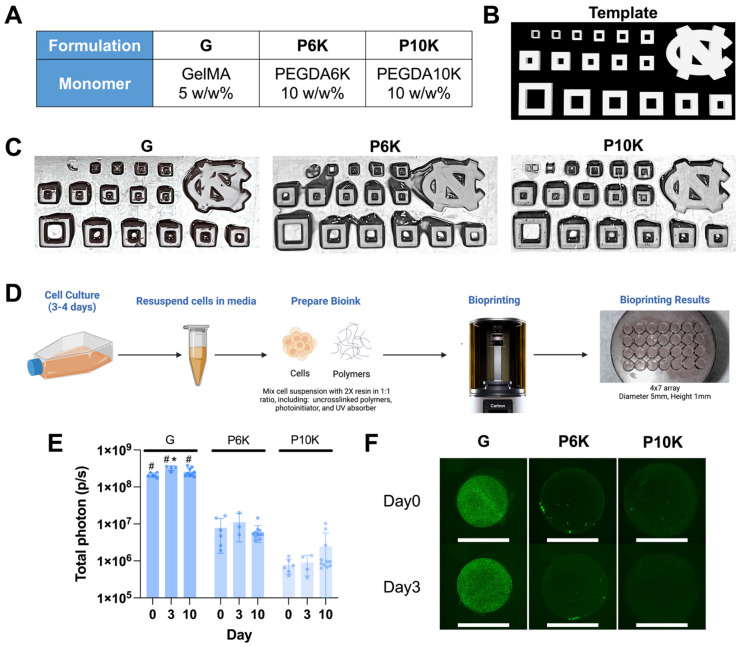
Evaluation of printability and in vitro bioprinted cell viability with single monomer resins. (**A**) Different resin formulations: **G**: 5 *w*/*w*% GelMA; **P6K**: 10 *w*/*w*% PEGDA6K; **P10K**: 10 *w*/*w*% PEGDA10K. All formulations are prepared with 0.25 *w*/*w*% LAP in mixed DPBS and DMEM media. (**B**) Printability test CAD design template. 1st row: Fixed hollow size: 1 mm. Strut width (mm, from left to right): 0.05, 0.1, 0.2, 0.3, 0.4, 0.5. UNC logo. 2nd row: Fixed hollow size: 1 mm. Strut width (mm, from left to right): 1, 0.9, 0.8, 0.7, 0.6. 3rd row: Fixed strut width: 1 mm. Hollow size (mm, from left to right): 3, 2, 1.8, 1.6, 1.4, 1.2. (**C**) Printing resolutions from different resin formulations **G**, **P6K**, **P10K**. (**D**) Scheme of general CLIP bioprinting strategy. (**E**) With selected design (Small disk, 5 mm in diameter, 1 mm in height), BLI signal from NHF1^GFP-FL^ bioprinting scaffolds with resin formulations **G**, **P6K**, **P10K**. (**F**) Representative images showing Day 3 GFP-expressing fibroblasts bioprinted in CLIP scaffolds with resin formulations G, P6K, P10K after bioprinting. (Scale bar = 5 mm). One-way ANOVA was performed to compare Day 3 and Day 10 BLI signals of G, P6K, and P10K scaffolds with Day 0 signals. *: For G, Day 3 BLI is significantly higher than Day 0 BLI, *p* = 0.0176. One-way ANOVA was performed for comparing differences between G, P6K, and P10K BLI signal on each day. #: For Day 0, 3, and 10, BLI signals of G scaffolds are all significantly higher than P6K and P10K, *p* < 0.0001. (**E**) Representative images showing Day 3 GFP-expressing fibroblasts bioprinted in CLIP scaffolds with resin formulations G, P6K, P10K after bioprinting. (Scale bar = 5 mm).

Based on the cytotoxicity screening results, we developed three initial resin formulations containing either: 5 wt% of GelMA (**G**), 10 wt% of PEGDA 6K (**P6K**), and 10 wt% of PEGDA 10K (**P10K**) (Figure 2A) for further evaluation.

We next evaluated the printability of these formulations by qualitatively assessing printing resolution and structural fidelity through visual inspection. Using computer-aided design (CAD), we created an array of test structures featuring void sizes ranging from 1 mm to 3 mm and strut widths ranging from 0.05 mm to 1 mm (Figure 2B). This design was printed on the Carbon S1 printer with each resin formulation, and representative images of the final parts are displayed in Figure 2C. Printing resolution was evaluated by assessing edge definition and fidelity to designed void sizes.

For formulation **G**, most printed scaffolds exhibited poor edge definition and designs with the finest strut widths (0.05 mm and 0.1 mm) failed to print entirely, indicating limited resolution. For formulation **P6K**, modest improvements in edge sharpness were observed, and while strut features down to 0.1 mm were occasionally printed, they were poorly resolved, and the 0.05 mm features remained unprintable. In contrast, formulation **P10K** demonstrated markedly improved print fidelity. Scaffolds printed with **P10K** exhibited sharper edges and strut widths down to 0.1 mm were printed with high resolution. However, even with **P10K**, the 0.05 mm strut features could not be fully resolved, suggesting that this dimension approaches the resolution limit for the CLIP S1 printer with these resin systems.

We then evaluated the influence of resin formulation on in vitro cell viability post-bioprinting. Human fibroblast NHF1^GFP-FL^ cells were mixed with each bioresin and bioprinted into solid disks (Figure 2D), and cell viability was tracked via BLI for up to 10 days (Figure 2E). On Day 0, **G** scaffolds showed significantly higher viability than **P6K** (27-fold) and **P10K** (270-fold) scaffolds. In **G** scaffolds, viability increased approximately 1.5-fold by Day 3, then plateaued through Day 10. In contrast, **P6K** and **P10K** scaffolds exhibited persistently lower viability throughout the time course. Representative GFP fluorescence images at Day 0 and Day 3 confirmed these trends (Figure 2F).

Overall, PEGDA-based materials alone (**P6K**, **P10K**) were suboptimal for cell-laden bioprinting, as they provided limited support for cell viability compared to the GelMA-based formulation. However, while GelMA offers superior bioactivity, its lower print fidelity relative to PEGDA restricts its utility for fabricating scaffolds with high structural precision. Therefore, further optimization of resin composition was necessary to achieve a balance between bioactivity and printing resolution.

To achieve a balance between bioactivity and printing resolution, we developed a blended resin formulation containing both GelMA and PEGDA 6K, referred to as **G-P6K**. PEGDA 6K was selected over PGEDA 10K due to solubility constraints when forming co-monomer solutions with GelMA. To further improve printability, the photoabsorber (PA) Ecamsule was incorporated into the formulation, resulting in **G-P6K-PA**.

To characterize the role of Ecamsule in the bioresin system, we performed supplementary optical and photocuring analyses.

UV-Vis absorption spectra were collected for LAP, Ecamsule, and a combined LAP + Ecamsule solution in PBS (Figure S1). The combined LAP + Ecamsule formulation retained spectral features from both individual components, supporting that Ecamsule contributes measurably to the optical absorption profile of the resin system.

To further evaluate the influence of photoabsorber incorporation on resin curing behavior, we used an in-house curing dosage measurement method to compare formulations with and without Ecamsule (Figure S2). In this assay, a patterned 3 × 3 light-intensity array was projected through a glass interface onto resin samples, and cured thickness measurements were used to derive curing-related parameters including dose-to-cure (Dc), light attenuation parameter (α), and estimated time required to cure a 100 µm feature. These measurements showed that Ecamsule altered curing characteristics and increased effective light attenuation, consistent with its role as a photoabsorber for modulating light penetration during CLIP photopolymerization.

Finally, photo-differential scanning calorimetry (Photo-DSC) analysis was performed to monitor photocuring kinetics and calculate double-bond conversion of G-P6K and G-P6K-PA. The extent of photocuring can be extracted from Photo-DSC by dividing the measured heat of polymerization (*DH*_P_) by the theoretical heat of the polymerization (*DH*_p,th_). Similarly, the rate of polymerization can be determined by dividing the heat flow (*Q*) by *DH*_p,th_. Under the UV exposure window corresponding to standard printing conditions, G-P6K reached a double-bond conversion of **60.7 ± 4.2%** at a rate of **0.0065 ± 0.00067 s^−1^**, while G-P6K-PA reached a double-bond conversion of **42.9 ± 4%** at a rate of **0.12 ± 0.026 s^−1^** (Figure S3). The photoabsorber-containing formulation, G-P6K-PA, exhibited slower photocuring kinetics and required a longer exposure window to reach less curing completion, consistent with reduced light penetration and attenuated photopolymerization following Ecamsule incorporation, further supporting that Ecamsule modulates the UV curing process within the bioresin system.

**Figure 3:**
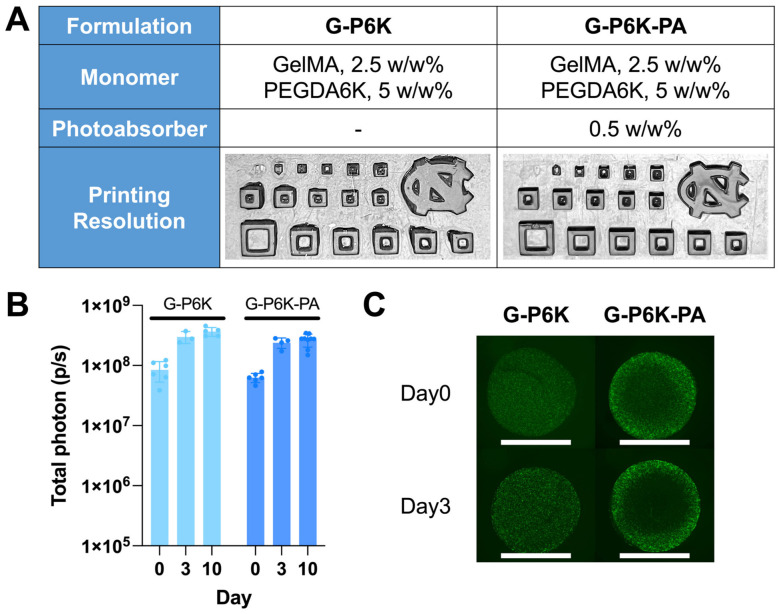
Evaluation of printability and in vitro bioprinted cell viability with dual-monomer resins. (**A**) Printing resolutions from different formulations, including **G-P6K**: 5 *w*/*w*% PEGDA6K, 2.5 *w*/*w*% GelMA; **G-P6K-PA**: 2.5 *w*/*w*% GelMA, 5 *w*/*w*% PEGDA6K, 0.5 *w*/*w*% PA. PA: Photoabsorber, Ecamsule. All formulations are prepared with 0.25 *w*/*w*% LAP in DPBS and DMEM 1:1 mixed solution. (**B**) With selected design (Small disk, 5 mm in diameter, 1 mm in height), BLI signal from NHF1^GFP-FL^ bioprinting scaffolds with resin formulations **G-P6K** and **G-P6K-PA**. (**C**) Representative images showing GFP-expressing fibroblasts bioprinted in CLIP scaffolds with resin formulations **G-P6K** and **G-P6K-PA**. (Scale bar = 5 mm).

After optical and curing characterization of Ecamsule, we then assessed the printability of **G-P6K** and **G-P6K-PA** using the resolution test array (Figure 3A). **G-P6K** exhibited sharp edge fidelity however struts with widths of 0.2 mm and 0.1 mm were only partially formed, and again the 0.05 mm design could not be printed. In contrast, **G-P6K-PA** demonstrated the highest printing resolution among all tested formulations. Scaffold edges were clean and well-defined, the 0.2 mm strut design was printed with excellent resolution, though the 0.1 mm design remained partially printed, and the 0.05 mm design still could not be resolved.

We then evaluated the influence of resin formulation on in vitro cell viability post-bioprinting, comparing **G-P6K** and **G-P6K-PA**. Human fibroblast NHF1^GFP-FL^ cells were mixed with each bioresin and bioprinted into solid disks. We first investigated in vitro cell viability using BLI over 10 days (Figure 3B). For **G-P6K** scaffolds, BLI signals increased 3.53-fold by Day 3 and 4.35-fold by Day 10. **G-P6K-PA** scaffolds showed a similar trend, with a 3.8-fold increase by Day 3 and a 4.24-fold increase by Day 10, despite slightly lower initial Day 0 BLI signal intensity compared to **G-P6K**. Fluorescence imaging further confirmed these results (Figure 3C). Cells in **G-P6K** scaffolds were distributed evenly and proliferated homogeneously. In contrast, **G-P6K-PA** scaffolds showed preferential accumulation of viable cells near the scaffold periphery, a trend that persisted over time. Nonetheless, both formulations supported high initial cell loading and sustained in vitro proliferation. Importantly, inclusion of the photoabsorber Ecamsule in **G-P6K-PA** did not adversely affect cell viability over time.

### 2.3. Impact of Material Composition on Cell In Vivo Persistence

**Figure 4:**
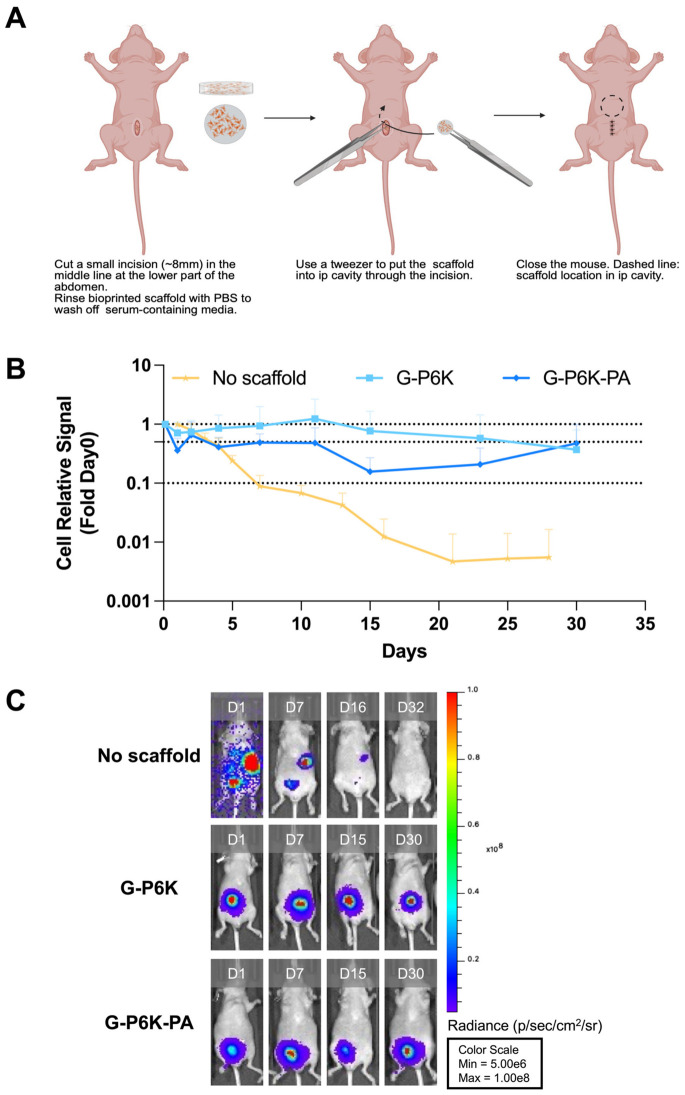
Impact of material composition on in vivo NHF1^GFP-FL^ persistence after intraperitoneal implantation. (**A**) Schematic illustration of surgical implantation of bioprinted scaffolds into the intraperitoneal cavity of mice. (**B**) Relative BLI signal of NHF1^GFP-FL^ in bioprinting scaffolds with different materials (**G-P6K**, n = 4; **G-P6K-PA**, n = 4) in the I.P. cavity, compared with free NHF1^GFP-FL^ injection (No scaffold, n = 4). A total of 12 mice was used in this study. Dashed lines indicate 1 time, 0.5 time, and 0.1 time relative cell signal fold change compared with Day 0 cell signal, from top to bottom. (**C**) In vivo bioluminescence images of the animals in (**B**).

We next evaluated whether bioprinted scaffolds could enhance in vivo cell persistence in the intraperitoneal (I.P.) cavity compared to direct injection. Immunodeficient nude mice were used to minimize immune-mediated clearance of human NHF1^GFP-FL^ cells. Scaffolds (5 mm diameter, 1 mm thickness) were printed using **G-P6K** or **G-P6K-PA** formulations and pre-cultured for 3 days to reach swelling equilibrium, eliminate unreacted components, and allow cell recovery prior to implantation. On the day of surgery, a midline incision was made to expose the I.P. cavity, one scaffold was placed into the cavity, and the incision was sutured closed (Figure 4A). For comparison, control groups received a direct injection 2 × 10^6^ NHF1^GFP-FL^ cells suspended in 300 µL of DPBS. Longitudinal BLI monitoring using the IVIS Spectrum system tracked cell persistence across all groups (Figure 4B,C).

Directly injected cells were not maintained within the IP cavity and were cleared rapidly, with 50% signal loss within 3 days and more than 90% loss by Day 7. In contrast, both **G-P6K** and **G-P6K-PA** scaffolds significantly prolonged cell persistence. **G-P6K** scaffolds initially showed a slight signal drop, followed by a rebound, reaching over 100% of the Day 0 signal by Week 2. By Day 25, 50% of the signal remained, and two mice retained detectable signal through Day 58 (Figure 4B and Figure S4). **G-P6K-PA** scaffolds, while also outperforming direct injection, showed a faster signal decline than **G-P6K**, with 50% loss by Day 7. The BLI signal then was followed by a rebound since Day 15, with 50% signal remaining by Day 30. More than 10% of the original signal persisted through Day 58. Two mice in the **G-P6K-PA** group died before study completion for unrelated reasons. Individual mouse BLI data are shown in Figure S1.

Together, these findings demonstrate that both **G-P6K** and **G-P6K-PA** bioprinted scaffolds significantly enhanced in vivo cell persistence in the I.P. cavity as compared to direct cell injection. Importantly, incorporation of the photoabsorber to enhance printability did not adversely affect in vitro or in vivo bioactivity.

### 2.4. Characterization of Bioprinted NHF1^GFP-FL^ G-P6K-PA Scaffolds

Based on high printing resolution, favorable in vitro biocompatibility, and elongated in vivo cell retention, the **G-P6K-PA** formulation represents an optimal bioresin for 3D bioprinting of scaffolds intended for intraperitoneal therapeutic cell delivery and was selected for further characterization.

**Figure 5:**
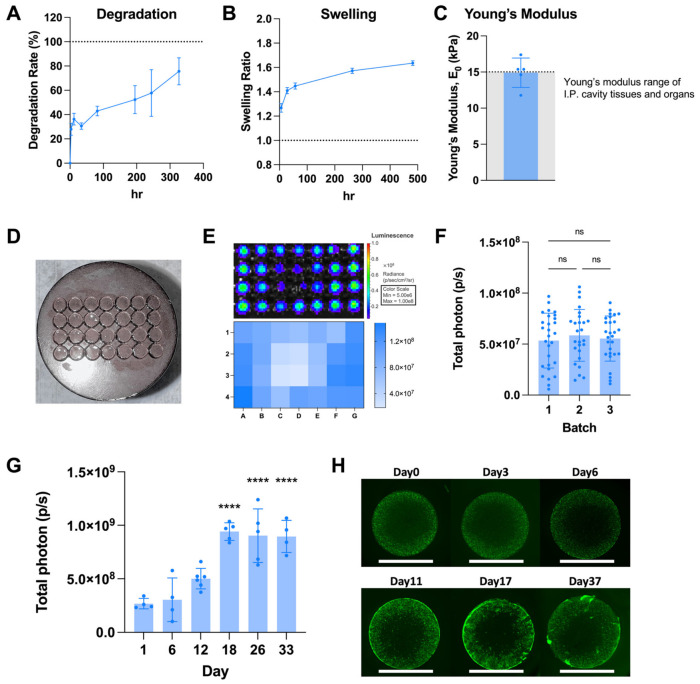
Characterization of 3D CLIP bioprinting NHF1^GFP-FL^ with G-P6K-PA. (**A**) Degradation rate of hydrogel printed with **G-P6K-PA**. (**B**) Swelling ratio of hydrogel printed with **G-P6K-PA**. (**C**) Young’s modulus of hydrogel printed with **G-P6K-PA**. (**D**) NHF1^GFP-FL^ is bioprinted with **G-P6K-PA** at a concentration of 5 × 10^6^ cells/mL into a 4 × 7 array of scaffolds (Diameter = 5 mm, Height = 1 mm) on the printing platform. Images of 3D bioprinted scaffolds for cell transplant. (**E**) IVIS image of all scaffolds within one batch of printing (**top**) and BLI signal distribution in a heat map (**bottom**). (**F**) NHF1^GFP-FL^ is bioprinted with **G-P6K-PA** at a concentration of 5 × 10^6^ cells/mL, n = 28 scaffolds per batch. 3 batches are printed separately. BLI signals from NHF1 in scaffolds from each batch are compared. (**G**) BLI signal from NHF1^GFP-FL^ bioprinting scaffolds with **G-P6K-PA** over time (n = 4~6). (**H**) Representative fluorescence image of GFP-positive NHF1 in bioprinted scaffolds with **G-P6K-PA** over time (brightness and contrast adjusted, scale bar = 5 mm). **** indicates *p* < 0.0001.

We first characterized the mechanical properties of CLIP 3D-printed acellular **G-P6K-PA** scaffolds. The degradation behavior of **G-P6K-PA** scaffolds by incubating them in collagenase solution and measuring dry weight at designated time points to calculate mass loss (Figure 5A). G-P6K-PA scaffolds exhibited ~75% mass loss over 14 days. Notably, this exceeded the expected degradation based on GelMA content alone (33.3% of total dry weight).

Swelling behavior was also characterized, as it influences the scaffold’s mechanical stability, porosity, and nutrient transport. **G-P6K-PA** scaffolds showed a swelling ratio of approximately 1.64 over 20 days (Figure 5B). The majority of swelling occurred within the first few days, stabilizing by Day 3 at a swelling ratio of 1.45.

Finally, we characterized the mechanical properties of **G-P6K-PA** scaffolds. Dog-bone shaped specimens were prepared, fully swelled, and subjected to tensile testing (Figure 5C). The scaffolds exhibited a Young’s modulus of 10–15 kPa, which falls within the range reported for native peritoneal tissues (1–15 kPa).

We then used G-P6K-PA to bioprint with NHF1^GFP-FL^ cells and assessed the consistency of cell loading across a single batch of bioprinted scaffolds, arranged in a 4 × 7 array (28 disks total; scaffold diameter = 5 mm, height = 1 mm) (Figure 5D,E). IVIS imaging (Figure 5E, top) and quantification of BLI signal from each scaffold (Figure 5E, bottom) revealed a spatial bias, with higher BLI signals in scaffolds printed near the edge of the platform compared to those in the center.

To address this variability, we increased the printing speed from 10 mm/h to 30 mm/h. This adjustment significantly improved printing outcomes: when printing at a density of 5 × 10^6^ cells/mL, the overall BLI signal increased by 3.88-fold, and the distribution of BLI signal across the array became more uniform (Figure S5).

To further validate the consistency and reproducibility of the CLIP 3D bioprinting process, we bioprinted three independent batches of **G-P6K-PA** scaffolds. BLI was used to quantify cell viability in each individual scaffold across batches (Figure 5F). No statistically significant differences were observed in mean BLI signals among batch 1 (5.34 ± 2.65 × 10^7^ p/s), batch 2 (5.85 ± 2.49 × 10^7^ p/s), and batch 3 (5.55 ± 2.17 × 10^7^ p/s), demonstrating reliable and reproducible cell loading across different production runs.

After confirming batch-level consistency, we next evaluated cell proliferation within bioprinted scaffolds over time. A single batch of 28 scaffolds was printed, and 4 to 6 scaffolds were randomly selected for BLI-based viability quantification on Days 1, 6, 12, 18, 26, and 33 (Figure 5G). The results revealed a steady increase in cell viability, reaching a 3.5-fold increase by Day 18. From Day 18 to Day 33, the BLI signals plateaued (3.4-fold on Day 26 and 3.3-fold on Day 33).

Fluorescence imaging of GFP-positive NHF1^GFP-FL^ cells supported the BLI data, showing increasing cell density over time (Figure 5H). Morphologically, cells transitioned from rounded shapes in the first week post-bioprinting to elongated, fibroblast-like structures by later time points. Notably, we observed stronger GFP signal intensity at the scaffold periphery compared to the center, indicating preferential cell growth at the edges.

### 2.5. Tuning Cell Loading in CLIP 3D-Bioprinted Scaffolds

**Figure 6:**
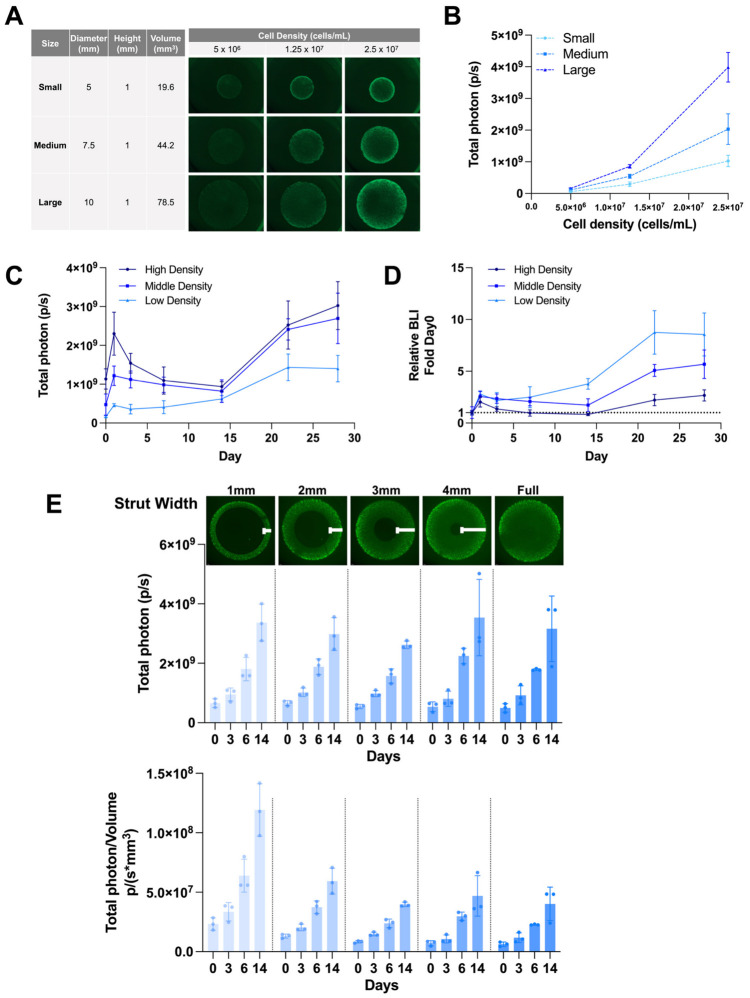
Tuning cell loading in CLIP 3D-bioprinted scaffolds. (**A**) Representative fluorescent images of GFP-positive NHF1s bioprinted with G-P6K-PA bioresin at various cell densities (Low Density: 5 × 10^6^ cell/mL, Middle Density: 1.25 × 10^7^ cell/mL, High Density: 2.5 × 10^7^ cell/mL) and different scaffold sizes (Small: diameter 5 mm, Medium: diameter 7.5 mm, Large: diameter 10 mm). (**B**) BLI signal of NHF1s bioprinted with G-P6K-PA at various cell densities and different scaffold sizes, corresponding to the data in panel (**A**). (**C**) Absolute longitudinal BLI signal of NHF1s bioprinted with G-P6K-PA at various cell densities. Scaffold sizes are small (diameter 5 mm) for all groups. (**D**) Relative longitudinal BLI signal of NHF1s bioprinted with G-P6K-PA at various cell densities. Scaffold sizes are small (diameter 5 mm) for all groups. (**E**) Representative fluorescent images of GFP-positive NHF1s bioprinted with G-P6K-PA at 5 × 10^6^ cell/mL with various strut width. Corresponding BLI signal over time (**top**) and BLI signal divided by scaffold volume (**bottom**) were displayed.

After establishing a consistent and reproducible CLIP 3D bioprinting protocol, we further evaluated scalability by varying cell loading densities, scaffold dimensions, and structural designs. Scaffolds were printed using **G-P6K-PA** resin with NHF1^GFP-FL^ cells at three different concentrations: low (5 × 10^6^ cells/mL), medium (1.25 × 10^7^ cells/mL), and high (2.5 × 10^7^ cells/mL), and across three scaffold sizes: small (5 mm), medium (7.5 mm), and large (10 mm) in diameter (Figure 6A). As expected, scaffolds with higher cell concentrations and larger dimensions produced brighter GFP signals and higher BLI intensities (Figure 6A,B and Figure S6A).

To further characterize the effects of cell density on post-printing behavior, we tracked both absolute and relative BLI signals in 5 mm scaffolds over time (Figure 6C,D and Figure S6B,C). While absolute BLI signals increased proportionally with initial cell density, the relative proliferation rate decreased. Specifically, low-density scaffolds exhibited the fastest growth, followed by medium-density and then high-density scaffolds. Interestingly, all cell density groups exhibited a tri-phasic growth pattern: an initial increase in BLI signal within the first 24 h, followed by a transient plateau or decline, and then a recovery phase with continued proliferation.

To explore the effects of scaffold design on cell loading efficiency and proliferation, we evaluated a series of ring-shaped scaffolds, all with a fixed 10 mm outer diameter and cell concentration (5 × 10^6^ cells/mL), but with varying strut thicknesses ranging from 1 mm to 5 mm (where 5 mm corresponds to a solid disk) (Figure 6E). Despite substantial differences in printed volume, the Day 0 BLI signals were similar across all designs (Figure 6E, top), indicating that thinner scaffolds, although containing less solid material, encapsulated a comparable number of viable cells. Furthermore, BLI signals steadily increased in all designs over a two-week period, indicating that these architectural variations did not impair in vitro cell proliferation.

To assess design-dependent cell-loading efficiency, we calculated printing efficiency by normalizing BLI signal to scaffold volume (p/s/mm^3^). The 1 mm strut scaffold exhibited the highest efficiency, achieving a 3.7-fold improvement over the solid scaffold on Day 0 and a 3.0-fold increase by Day 14 (Figure 6E, bottom). GFP fluorescence imaging confirmed these results. Notably, except for the 1 mm design, all other scaffold types displayed uneven cell distribution, with brighter fluorescence at the scaffold edges and diminished signal closer to the center.

### 2.6. Extending Bioprinting to Stem Cells

Stem cells hold significantly greater clinical potential due to their ability to secrete regenerative and anti-cancer factors. Previous studies have shown that therapeutic stem cells secreting anti-cancer reagents can be effective against I.P. tumors such as ovarian cancer [2,67,68,69]. However, rapid clearance from the peritoneal cavity remains a major challenge for stem cell therapies.

To evaluate the versatility of our CLIP bioprinting platform, we extended our studies to three different human stem cell types: (1) human mesenchymal stem cells (hMSC^mCherry-FL^), (2) human neural stem cells (HB1.F3^GFP-FL^), and (3) a lab-derived spheroidal human induced neural stem cell line (hiNeuroS^mCherry-FL^). Each cell type was suspended in **G-P6K** resin at appropriate densities: 2.5 × 10^6^ cells/mL for hMSC, and 1 × 10^7^ cells/mL for HB1.F3 and hiNeuroS. Cell viability was assessed via BLI, and fluorescence imaging was used to monitor cell distribution within the scaffolds.

**Figure 7:**
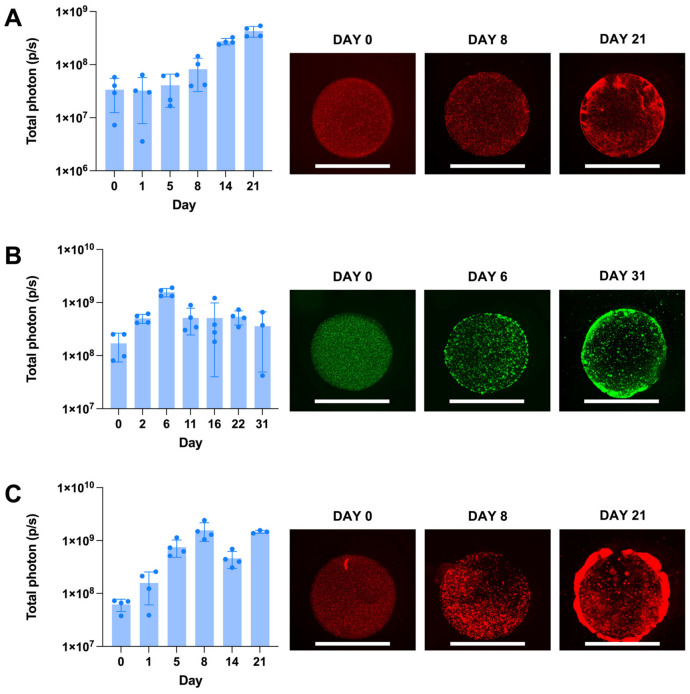
Application of CLIP Bioprinting on stem cells. (**A**) BLI signal of 1.25 × 10^6^ cell/mL human mesenchymal stem cell line hMSC^mCherry-FL^ bioprinting scaffolds with G-P6K, and representative mCherry fluorescent images over time. Scale bar = 5 mm. (**B**) BLI signal of 5 × 10^6^ cell/mL human neural stem cell line HB1.F3^GFP-FL^ bioprinting scaffolds with G-P6K, and representative GFP fluorescent images over time. Scale bar = 5 mm. (**C**) BLI signal of 5 × 10^6^ cell/mL human induced spheroidal neural stem cell hiNeuroS^mCherry-FL^ bioprinting scaffolds with G-P6K, and representative mCherry fluorescent images over time. Scale bar = 5 mm.

hMSC^mCherry-FL^ cells demonstrated excellent biocompatibility with the bioprinting process (Figure 7A). BLI signals increased steadily over 3 weeks, reaching a 12-fold increase by Day 21. Fluorescence imaging confirmed this trend.

BLI data for HB1.F3^GFP-FL^ cells revealed a 9-fold increase in signal within the first 6 days, followed by a decline and plateau at approximately 3-fold by Day 31 (Figure 7B). GFP imaging confirmed this trend.

hiNeuroS^mCherry-FL^ cells exhibited a more dynamic growth pattern (Figure 7C). BLI signals increased 25-fold by Day 8, dropped to 7-fold by Day 14, and recovered to 24-fold by Day 21. Fluorescence imaging showed that although the spheroidal cells were initially dispersed into smaller aggregates or single cells during resin mixing, they re-aggregated into spheroids. Over time, these cells formed large clusters both within the scaffold center and at its periphery.

## 3. Discussion

This study successfully developed and validated a CLIP-based 3D bioprinting platform for fabricating cell-laden hydrogel scaffolds capable of extending cell persistence in the challenging I.P. environment. Our primary contribution is the establishment of a robust biofabrication toolkit, encompassing an optimized bioresin, validated printing protocols, and clear design rules, that addresses a critical delivery barrier for regional cell therapies. By systematically characterizing the interplay between material properties, scaffold architecture, and biological outcomes, we have laid the groundwork for engineering the next generation of I.P. implantable cell delivery systems (Table S1).

The physical and chemical properties of the scaffold were key determinants of its success. The final **G-P6K-PA** formulation yielded an initial Young’s modulus of 10–15 kPa, closely matching that of native peritoneal tissue (1–15 kPa). This mechanical mimicry is critical, as literature suggests that biomaterial stiffness is a key regulator of the foreign body response, with tissue-matched softness promoting a more favorable, less fibrotic host response. Importantly, while very soft hydrogels (<1 kPa) may further minimize FBR, they often lack the mechanical integrity required for surgical handling and reliable intraperitoneal placement. Our formulation, therefore, represents an optimized balance between biocompatibility and practical surgical utility. The swelling behavior of our scaffolds also has important implications for clinical translation. The majority of swelling occurred within the first three days, suggesting that a brief pre-conditioning period prior to implantation could minimize further volumetric changes in vivo, thereby improving implant stability and reducing the risk of mechanical irritation. Furthermore, the observed degradation of **G-P6K-PA** scaffolds (~75% mass loss over 14 days) exceeded the theoretical contribution of the GelMA component alone (33.3%), suggesting a complex degradation mechanism involving the release of PEGDA segments following enzymatic breakdown of the GelMA backbone. This finding demonstrates that blending degradable and non-degradable polymers provides a powerful strategy for tuning scaffold degradation profiles to match specific therapeutic timelines.

The most compelling finding of this work was the dramatic extension of in vivo cell persistence. The scaffolds transformed a transient cell delivery, where free cells were cleared in under a week, into a durable one, with detectable cell signals lasting for nearly two months in some animals. This represents an order-of-magnitude improvement in functional duration. We attribute this profound effect to the protective niche provided by the hydrogel matrix, which likely shields encapsulated cells from the harsh mechanical and immunological clearance mechanisms of the I.P. cavity, including peritoneal fluid shear and macrophage-mediated phagocytosis.

Beyond material composition, we demonstrated that printing parameters and cell density need to be considered to control cell loading and growth dynamics. The significant improvement in cell viability observed at higher printing speeds (3.88-fold increase at 30 mm/h vs. 10 mm/h) is likely attributable to reduced cumulative UV exposure, which minimizes direct UV damage and cytotoxicity from generated free radicals. This finding has important implications for process optimization, suggesting that maximizing printing speed within the constraints of print fidelity is a key strategy for improving bioprinting outcomes. The inverse relationship between initial cell density and relative proliferation rate suggests that high-density scaffolds may approach the threshold for contact inhibition immediately after bioprinting. This finding has practical implications for therapeutic dosing: while higher initial cell loading can be achieved by increasing cell concentration, the long-term cell number may be constrained by density-dependent growth limits.

We also observed reduced cell viability in the central regions of scaffold designs, accompanied by preferential cell growth at the periphery. Analysis of scaffold architectures (Figure 6E) further revealed that thinner structures exhibited more uniform cell distribution and higher cell-loading efficiency, whereas thicker designs showed pronounced spatial heterogeneity. This trend is consistent with diffusion-limited transport of oxygen and nutrients in 3D hydrogel systems, where increased structural thickness leads to longer diffusion distances and reduced mass transport to the scaffold core. In contrast, thinner architectures shorten diffusion pathways and improve nutrient accessibility, supporting more uniform cell viability. These findings highlight the importance of scaffold architecture, particularly strut thickness and internal geometry, as key design parameters for optimizing mass transport and long-term cell performance. However, the uneven cell distribution observed within the scaffolds may result from active cell migration, localized proliferation, and/or spatial redistribution; the current endpoint imaging does not distinguish among these mechanisms. Future studies incorporating live-cell tracking or quantitative spatial localization analysis would help clarify the relative contributions of these processes.

The successful bioprinting of three distinct stem cell types, including mesenchymal, neural, and induced neural stem cells, demonstrates the versatility of our CLIP platform. Notably, HB1.F3 cells, typically cultured as adherent monolayers, spontaneously formed 3D clusters within the bioprinted scaffolds. Similarly, hiNeuroS cells, initially dispersed during resin mixing, successfully re-aggregated into spheroids. These observations indicate that the CLIP bioprinting process does not irreversibly disrupt stem cell behavior and that the scaffold matrix supports their natural growth patterns, providing a favorable 3D microenvironment.

While this study establishes a robust CLIP-based bioprinting platform for intraperitoneal cell delivery, several limitations and future directions should be acknowledged. As a platform-validation study, we did not incorporate therapeutic cells or evaluate efficacy in a disease model. The next logical step is to load these scaffolds with therapeutic cell populations and evaluate their therapeutic impact in relevant peritoneal disease models. Future studies should also assess how the bioprinting process affects cell phenotype, gene expression, differentiation capacity, secretory activity, and migratory behavior, all of which are critical parameters for clinical translation.

It is also important to note that the mechanical characterization performed in this study represents an initial baseline tensile assessment. Additional mechanical characterization, including compressive testing and evaluation at multiple degradation time points, would provide valuable insight into how scaffold mechanics evolve during degradation and implantation, and should be incorporated in future studies.

In addition, further optimization of scaffold design should incorporate the unique physiological constraints of the intraperitoneal environment. The peritoneal cavity is a relatively avascular and diffusion-limited space, where nutrient and oxygen transport to implanted constructs primarily depends on passive diffusion through peritoneal fluid. Therefore, scaffold thickness, porosity, and internal architecture are expected to strongly influence long-term cell survival and therapeutic function. Consistent with this consideration, we observed reduced cell viability in the central regions of thicker scaffold structures, with preferential cell growth at the periphery, suggesting diffusion-limited transport within the hydrogel matrix. These findings highlight the importance of designing thin or porous scaffold architectures to reduce diffusion distance and improve mass transport.

The high-resolution capability of CLIP bioprinting provides a unique opportunity to engineer such environment-informed scaffold designs. For example, future intraperitoneal scaffold architectures may draw inspiration from clinically used porous mesh implants, such as hernia repair meshes, which are designed with micron-scale filaments and millimeter-scale pores to balance tissue integration, mechanical stability, and reduced fibrotic encapsulation. In this context, achieving tens-of-micrometers-scale printing resolution is important not only for demonstrating manufacturing precision but also for enabling future fabrication of mesh-like, porous, and thin implant geometries tailored to the intraperitoneal environment.

Finally, detailed histological characterization of the host response to implanted scaffolds will be essential to fully understand implant–tissue interactions, in vivo therapeutic cell fate, inflammation, fibrotic encapsulation, and foreign body response. These studies will be important for defining how scaffold material properties and architecture influence long-term biocompatibility and translational potential in the intraperitoneal cavity.

## 4. Materials and Methods

### 4.1. Materials, Reagents, and Cell Lines

Gelatin Methacrylate (GelMA, Type A, 300 Bloom, Porcine Gelatin, degree of methacrylation = 45–65%, #VL3500000502), Polyethylene Glycol Diacrylate (PEGDA) with molecular weights of 6000 (PEDGA6K, #5339-1GM) and 10,000 (PEDGA10K, #5340-1GM) were all purchased from Advanced Biomatrix (Carlsbad, CA, USA). UV radical initiator lithium phenyl-2,4,6-trimethylbenzoylphosphinate (LAP, #L0290) was purchased from TCI Chemicals (Portland, OR, USA). Photoabsorber Ecamsule disodium (Cas No.: 90458-75-6, #GC61828) was purchased from GlpBio (Montclair, CA, USA). Dulbecco’s phosphate-buffered saline (DPBS, #14190-144) was purchased from Gibco (Grand Island, NY, USA). D-Luciferin potassium salt (#122799) was purchased from Revvity (Waltham, MA, USA). Type I collagenase from *Clostridium histolyticum* with ≥125 CDU/mg solid (#C0130-100MG) was purchased from Sigma-Aldrich (St. Louis, MO, USA). For the animal study, absorbable suture Med Vet International SUTURE VICRYL 4-0 CLEAR (J422H, #50-118-0841) was purchased from Fisher Scientific (Waltham, MA, USA).

NHF1 cells were obtained from W. Kauffman (University of North Carolina School of Medicine) and were hTERT-immortalized. HB1.F3.CD cells were obtained from Dr. Karen Aboody (City of Hope National Medical Center) and generated as previously described [70]. Both cell lines were cultured using Dulbecco’s Modified Eagle Medium (DMEM, #11995-065. Gibco), containing 10% fetal bovine serum (FBS, #35-015-CV. Corning, Corning, NY, USA) and 1% penicillin-streptomycin (Pen Strep, 10,000 units penicillin and 10 mg streptomycin/mL, #15140-122. Gibco), hereby referred to as standard culture medium.

Adipose-derived human mesenchymal stem cells (hMSC, PCS-500-011) were purchased from ATCC (Manassas, VA, USA). hMSC cells were cultured in Dulbecco’s Modified Eagle Medium with low glucose and GlutaMAX™ supplement (Gibco, #10567-014), containing 10% FBS and 1% Pen Strep.

All cell lines were incubated in 5% CO_2_ at 37 °C and passaged periodically using 0.05% Trypsin-EDTA (Gibco, #25300-054) and centrifugation at 1000× *g* for 5 min.

### 4.2. hiNeuroS Generation

hiNeuroS cells were generated as previously described [71]. hiNeuroS cells are cultured in ReNcell media (Sigma-Aldrich, #SCM005) and supplemented with doxycycline (#631311. Takara, San Jose, CA, USA), epidermal growth factor (EGF, #50-990-731. Fisher Scientific), and fibroblast growth factor (FGF. Fisher Scientific, #50-104-7691) every other day. The hiNeuroS cell line was incubated in 5% CO_2_ at 37 °C and passaged periodically using centrifugation. Before use, hiNeuroS cells were dissociated into a single-cell suspension with Accutase (Sigma Aldrich, #A6964-100ML).

### 4.3. Lentiviral Transduction

Cells were treated with 8 µg/mL polybrene and the lentiviruses for 24 h at 37 °C and 5% CO_2_. NHF1 and HB1.F3 cells were transduced with lentiviruses encoding for green fluorescent protein (GFP) and firefly luciferase (Fluc), hereby referred to as NHF1^GFP-FL^ and HB1.F3^GFP-FL^. hMSC and hiNeuroS cells were transduced with lentiviruses encoding mCherry (mCh) and Fluc, hereby referred to as hMSC^mCherry-FL^, hiNeuroS^mCherry-FL^. All lentiviruses were purchased from the Duke Viral Vector Core.

### 4.4. Resin Toxicity Screening

20,000 NHF1^GFP-FL^ cells were seeded in a black-walled, clear-bottomed 96-well plate one day before. Materials were prepared into different concentrations with the standard culture medium for NHF1 and added to NHF1 cells. To explore the impact of UV exposure, cells were exposed to 365 nm UV light (100% intensity) for 60 s. The cells were then cultured within the resin in 5% CO_2_ at 37 °C for 1 h. Cells were then treated with 10 uL luciferin solution (15 mg/mL D-luciferin dissolved in 1X DPBS) for 5 min prior to bioluminescence imaging using the in vivo imaging system (IVIS) Spectrum. Viability for each treatment group was calculated as the BLI signal for each treatment group divided by the average BLI signal from non-treated cells.

### 4.5. CLIP 3D Bioprinting

CLIP bioprinting was performed using the S1 CLIP prototype printer (Carbon), utilizing a 385 nm LED UV light source. The cylindrical scaffold designs were created in TinkerCAD (https://www.tinkercad.com/ (accessed on 2 March 2026)) and exported as STL files, which were sliced at 1 μm using the Carbon printing software. For G-P6K-PA formulation, a 2X concentration resin was prepared first by mixing 5 *w*/*w*% GelMA, 10 *w*/*w*% PEGDA6K, 0.5 *w*/*w*% LAP, 1 *w*/*w*% Ecamsule, and 83.5 *w*/*w*% DPBS at 55 °C and 500 RPM until fully dissolved. Next, the 2X concentration resin was mixed thoroughly in a 1:1 volume ratio with 0.75~1 mL 1 × 10^7^~5 × 10^7^ cells/mL NHF1 cell suspension in standard culture medium by pipetting, resulting in final bioresin concentrations of 2.5% GelMA, 5% PEGDA6K, 0.25% LAP, 0.5% Ecamsule, and 5 × 10^6^~2.5 × 10^7^ cells/mL in 1.5~2 mL total bioresin mixture. The bioresin mixture was then added to the printer cassette, and bubbles were carefully removed from the bioresin prior to initiating 3D printing. Scaffolds were printed at a continuous speed of 10 mm/h and light intensity of 12 mW/cm^2^. Following 3D production, excess resin was removed from the scaffolds via gentle washing with deionized water, and scaffolds were immediately placed individually into 48-well plates containing 1.5 mL fresh standard culture medium per well. Scaffolds were cultured at 37 °C and 5% CO_2_ with media changes occurring at 24 h post-printing, and every subsequent 48 h. All the other resin formulations and cells were prepared using the same procedure.

### 4.6. Printability

An array of hollow scaffolds with different hollow sizes (ranging from 1 mm to 3 mm) and strut widths (ranging from 0.05 mm to 1 mm) was designed on TinkerCad. Different formulations were prepared as stated in the previous section to print the scaffold array. Printing parameters were optimized for each resin to ensure the best printing results for each resin. Pictures were taken for the printed results of each resin.

### 4.7. Photo-Differential Scanning Calorimetry (Photo-DSC) Study

Photo-DSC experiments were performed with an Omnicure s2000 (Excelitas, Pittsburgh, PA, USA) light source equipped with a 320~390 nm lens filter. The light was calibrated using a Dymax ACCU-CAL™ 50 radiometer (Dymax, Torrington, CT, USA). To mimic real CLIP 3D printing UV light projecting process for each resin, the light was calibrated to 1 mW/cm^2^ and conversion data was taken for G-P6k for 122 s; the light was calibrated to 12 mW/cm^2^ and conversion data was taken for G-P6K-PA for 376 s. Double bond conversion was determined by dividing the experimental heat of polymerization (*H*_p_) by the theoretical enthalpy (Δ*H*_th_) and the rate of polymerization (*R*_p_) was determined by dividing heat flow (Q) by (Δ*H*_th_).

### 4.8. Degradation Study

Scaffolds were cured in molds with dimensions of 12.5 mm × 10 mm × 5 mm (L × W × H) using 100% intensity 365 nm UV light. Scaffolds were then taken out and put into INCU-Line digital incubators (VWR, Radnor, PA, USA) set at 37 °C to dry. Initial scaffold dry weight was measured (W_0,D_). 5 mL of 1.5 unit/mL collagenase in DPBS solution was added to scaffolds. Scaffolds were degraded at 37 °C under agitation at 100 rpm on an incubating orbital shaker (VWR, #3500I). Collagenase solution was changed every 3 days. Scaffolds were washed and collected at certain time points, and then dried in an oven to measure the dry weight (W_T,D_) after degradation. Degradation rate (%) over time was calculated using the formula:

Degradation rate (%) = (W_0,D_ − W_T,D_)/W_0,D_ × 100%

### 4.9. Swelling Test

Scaffolds were cured in molds with dimensions of 12.5 mm × 10 mm × 5 mm (L × W × H) using 100% intensity 365 nm UV light. Initial scaffold wet weight was measured (W_0,W_). Each scaffold was incubated in 5 mL DPBS and was swelled at 37 °C under agitation at 100 rpm on an incubating orbital shaker. The DPBS solution was changed every 3 days. Wet scaffold weight was measured at different time points (W_T,W_).

Swelling Ratio = (W_T,W_ − W_0,W_)/W_0,W_

### 4.10. Modulus Test

Hydrogel think sheets (4 cm × 3.5 cm × 1 mm, L × W × H) were CLIP 3D printed using G-P6K-PA and fully swelled before the modulus test. Dogbone-shaped hydrogel scaffolds were then cut from the 3D-printed sheets using a mold with bridge dimensions 12 mm  ×  2 mm. Samples were loaded into an RSA-G2 dynamic mechanical analysis (DMA) system (TA Instruments) and stretched uniaxially at a constant strain rate of 0.005 s^−1^ until the breaking point.

### 4.11. In Vitro Bioluminescence Viability Assay

Bioluminescence was used to compare cell densities in all scaffolds printed with firefly luciferase-expressing cells (NHF1^GFP-FL^, hMSC^mcherry-FL^, HB1.F3^GFP-FL^, hiNeuroS^mCherry-FL^). Shortly after printing, the scaffolds were placed in a black-walled, clear-bottomed 24-well plate in 1 mL media. In total, 100 μL of 15 mg/mL D-luciferin solution was then added to each well. The samples were incubated in the luciferin solution for 20 min prior to bioluminescence imaging using the IVIS Spectrum. To measure NHF1^Fluc^ viability in the scaffolds over time, scaffolds cultured for various time points between 0 and 28 days were incubated in luciferin and imaged in the same manner.

### 4.12. Free NHF1^GFP-FL^ Cell Injection into Mouse Intraperitoneal Cavity and Persistence Imaging

Animal studies were approved by the University of North Carolina at Chapel Hill’s Animal Care and Use Committee (IACUC) (IACUC ID 25-016). Female, athymic nude mice (Animal Studies Core, University of North Carolina at Chapel Hill) aged 6–8 weeks and weighing 18–23 g were used for all studies. The animals were first anesthetized using 3% inhaled isoflurane. NHF1^GFP-FL^ cells were collected and resuspended in a final concentration of 2 × 10^6^ cells in 300 μL DPBS. In total, 2 × 10^6^ cells in 300 μL DPBS were then injected into the I.P. cavity of the mice. Serial BLI imaging was performed for two months using the IVIS spectrum with 150 mg/kg intraperitoneal injection of 15 mg/mL D-luciferin in 1X DPBS and 25 min as the waiting time before imaging.

### 4.13. In Vivo NHF1^GFP-FL^ Bioprinted Scaffolds Implantation into Mouse Intraperitoneal Cavity

Animal studies were approved by the University of North Carolina at Chapel Hill’s Animal Care and Use Committee (IACUC) (IACUC ID 25-016). Female, athymic nude mice (Animal Studies Core, University of North Carolina at Chapel Hill) aged 6–8 weeks and weighing 18–23 g were used for all studies. 3 days prior to the implantation surgery, bioprinted scaffolds (Diameter = 5 mm, Height = 1 mm) were prepared as described above and cultured for 3 days. On the day of implantation, animals were anesthetized using 3% inhaled isoflurane, then placed into a stereotactic frame. The surgical site was prepared using antiseptics, betadine and 70% isopropyl alcohol. A midline incision was made to expose the intraperitoneal cavity. Scaffolds were inserted at the site of the incision, after which the wound was closed using standard surgical techniques.

Pre-operative pain management was performed using subcutaneous injection of 5 mg/kg meloxicam moments prior to surgery.

Post-operative pain management was performed using subcutaneous injection of 5 mg/kg meloxicam directly, 24h and 48 h post-surgery.

### 4.14. In Vivo NHF1^GFP-FL^ Persistence in Bioprinted Scaffolds

Serial BLI imaging was performed 2 to 3 times every week for two months using the IVIS spectrum with 150 mg/kg intraperitoneal injection of 15 mg/mL D-luciferin in 1X DPBS and 25 min as the waiting time before imaging.

### 4.15. Statistical Analysis

All results are presented as mean ± standard error of the mean. Modulus data, batch-to-batch consistency data, in vitro bioluminescence viability data were analyzed via one-way ANOVA with Tukey’s Multiple Comparisons test. In vivo persistence data was analyzed via Student’s *t* test. For all analyses, ns indicates not significant, * indicates *p* < 0.05, ** indicates *p* < 0.01, *** indicates *p* < 0.001, and **** indicates *p* < 0.0001. Statistical analyses were conducted using Prism GraphPad (version 10).

## 5. Conclusions

In conclusion, we have developed a versatile and scalable CLIP-based 3D bioprinting platform for the fabrication of cell-laden hydrogel scaffolds tailored for intraperitoneal delivery. By systematically optimizing the bioresin formulation, printing parameters, and scaffold architecture, we established clear design rules that enable precise control over material properties and biological outcomes. The resulting scaffolds demonstrated tissue-matched mechanical properties, tunable degradation, and, most critically, the ability to extend in vivo cell persistence by an order of magnitude. The platform’s versatility was further confirmed by its compatibility with multiple clinically relevant stem cell types. This work provides a robust biofabrication strategy to overcome a key delivery barrier in regional cell therapy and lays the foundation for engineering the next generation of implantable living drug delivery systems.

## Figures and Tables

**Table 1:** Summary of Clinical and Preclinical Applications of Intraperitoneal Cell Therapy (as of 24 February 2026).

Disease/Indication	Cell Type in Clinical Trials (Representative Study Number)	Cell Type in Preclinical Studies (Key Reference)
**Cancer**		
Ovarian Cancer	CAR-T (NCT07181720) CAR-M (NCT04660929) NK (NCT06321484) MSC-INFβ (NCT02530047) γδ T Cells (Pilot, UMIN-CTR UMIN000015233 [5])	NSC-nanoparticle Conjugate [2] Polymer-encapsulated RPE-IL2 [6] Cytokine-Induced Killer (CIK) Cells [7] iPSC-Derived NK Cells [8]
Colorectal/Colon Cancer	CAR-T (NCT07179692)	CAR-NK cells [9]
Gastric Cancer	CAR-T (NCT07179692) γδ T Cells (Pilot, UMIN-CTR UMIN000004130 [10]) Tumor-Associated Lymphocyte (TALs) [11]	Cytokine-Induced Killer (CIK) Cells [12] CAR-NK Cells [13]
Esophagogastric Cancer	CAR-T (NCT06623396) CAR-M (NCT04660929)	
**Regenerative Cell** **Replacement**		
Pediatric Acute Liver Failure	Alginate Microbeads Encapsulated Hepatocyte and Mesenchymal Stromal Cells (NCT05491135, “HELP”- first-in-huma trial [14])	Micro-encapsulated Hepatocyte [15]
Type 1 Diabetes	Alginate-Polylysine Microcapsules Encapsulated Human Islet Cells (first-in-human trial [16])	Encapsulated Islet Cells [17]
**Inflammatory** **& Autoimmune Disease**		
Crohn’s Disease	Tolerogenic Dendritic Cells (EudraCT number 2007-003469-42)	MSCs [18]
Colitis	-	MSCs [19] Alternatively Activated Macrophages [20]
Necrotizing Enterocolitis (NEC)	-	Amniotic Fluid Stem Cells (AFSCs) [21]
Liver Fibrosis		Microencapsulated MSCs [22]
Peritoneal Fibrosis (Dialysis)	-	MSCs [23]
Bladder Fibrosis	-	MSCs [24]

## Data Availability

The raw data supporting the conclusions of this article will be made available by the authors on request.

## Supplementary Materials

The following supporting information can be downloaded at: https://www.mdpi.com/article/10.3390/molecules31111958/s1, Figure S1: UV-Vis absorption spectra of LAP, Ecamsule, and combined LAP + Ecamsule solutions; Figure S2: Effect of photoabsorber incorporation on resin curing behavior; Figure S3: Evaluation of double bond conversion and rate of photocuring via Photo-DSC; Figure S4: Impact of material composition on in vivo cell persistence after intraperitoneal implantation; Figure S5: Increasing the printing speed leads to significantly higher cell viability after bioprinting; Figure S6: Tuning cell loading in CLIP 3D-bioprinted scaffolds; Table S1: Summary of resin formulation variables and key performance outcomes.

## Abbreviations

The following abbreviations are used in this manuscript:

BLI	Bioluminescence
CAD	Computer-Aided Design
CAR-M:	Chimeric Antigen Receptor-Macrophages
CAR-NK	Chimeric Antigen Receptor-Natural Killer Cells
CAR-T	Chimeric Antigen Receptor-T Cells
CLIP	Continuous Liquid Interface Production
DDS	Drug Delivery Systems
ECM	Extracellular Matrix
EudraCT	European Union Drug Regulating Authorities Clinical Trials Database
FBR	Foreign Body Response
GelMA	Gelatin Methacrylate
hiNeuroS	Spheroidal Human Induced Neural Stem Cell Line
I.P.	intraperitoneal
iPSC	Induced Pluripotent Stem Cell
LAP	Lithium Phenyl-2,4,6-Trimethylbenzoylphosphinate
MSC	Mesenchymal Stem Cells
MSC-INFβ	MSC-Infβ:Mesenchymal Stem Cells Secreting Infβ
NK	Natural Killer Cells
NSC	Neural Stem Cells
PA	Photoabsorber
PEGDA	Polyethylene Glycol Diacrylate
Photo-DSC	Photo-Differential Scanning Calorimetry
RPE-IL2	Human Retinal Pigmented Epithelial Cells Producing Interleukin-2
UMIN-CTR	University Hospital Medical Information Network Clinical Trials Registry, Japan

## References

[1] Ding Y., Wang Y., Hu Q. (2022). Recent advances in overcoming barriers to cell-based delivery systems for cancer immunotherapy. Exploration.

[2] Cao P., Mooney R., Tirughana R., Abidi W., Aramburo S., Flores L., Gilchrist M., Nwokafor U., Haber T., Tiet P. (2017). Intraperitoneal administration of neural stem cell–nanoparticle conjugates targets chemotherapy to ovarian tumors. Bioconjugate Chem..

[3] Perelló-Trias M.T., Serrano-Munoz A.J., Rodríguez-Fernández A., Segura-Sampedro J.J., Ramis J.M., Monjo M. (2024). Intraperitoneal drug delivery systems for peritoneal carcinomatosis: Bridging the gap between research and clinical implementation. J. Control. Release.

[4] Ceelen W.P., Flessner M.F. (2010). Intraperitoneal therapy for peritoneal tumors: Biophysics and clinical evidence. Nat. Rev. Clin. Oncol..

[5] Abe Y., Kobayashi H., Kanno T., Akizawa Y., Ishitani K., Hashimoto K. (2024). Intraperitoneal Injection of Expanded Gamma Delta T Cells with Zoledronate: A Pilot Analysis of Seven Patients with Ovarian Cancer. Ann. Cancer Res. Ther..

[6] Nash A.M., Jarvis M.I., Aghlara-Fotovat S., Mukherjee S., Hernandez A., Hecht A.D., Rios P.D., Ghani S., Joshi I., Isa D. (2022). Clinically translatable cytokine delivery platform for eradication of intraperitoneal tumors. Sci. Adv..

[7] Chan J.K., Hamilton C.A., Cheung M.K., Karimi M., Baker J., Gall J.M., Schulz S., Thorne S.H., Teng N.N., Contag C.H. (2006). Enhanced killing of primary ovarian cancer by retargeting autologous cytokine-induced killer cells with bispecific antibodies: A preclinical study. Clin. Cancer Res..

[8] Hermanson D.L., Bendzick L., Pribyl L., McCullar V., Vogel R.I., Miller J.S., Geller M.A., Kaufman D.S. (2016). Induced pluripotent stem cell-derived natural killer cells for treatment of ovarian cancer. Stem Cells.

[9] Xiao L., Cen D., Gan H., Sun Y., Huang N., Xiong H., Jin Q., Su L., Liu X., Wang K. (2019). Adoptive transfer of NKG2D CAR mRNA-engineered natural killer cells in colorectal cancer patients. Mol. Ther..

[10] Wada I., Matsushita H., Noji S., Mori K., Yamashita H., Nomura S., Shimizu N., Seto Y., Kakimi K. (2014). Intraperitoneal injection of in vitro expanded Vγ9Vδ2 T cells together with zoledronate for the treatment of malignant ascites due to gastric cancer. Cancer Med..

[11] Kono K., Takahashi A., Ichihara F., Amemiya H., Iizuka H., Fujii H., Sekikawa T., Matsumoto Y. (2002). Prognostic significance of adoptive immunotherapy with tumor-associated lymphocytes in patients with advanced gastric cancer: A randomized trial. Clin. Cancer Res..

[12] Du X., Jin R., Ning N., Li L., Wang Q., Liang W., Liu J., Xu Y. (2012). In vivo distribution and antitumor effect of infused immune cells in a gastric cancer model. Oncol. Rep..

[13] Cao B., Liu M., Huang J., Zhou J., Li J., Lian H., Huang W., Guo Y., Yang S., Lin L. (2021). Development of mesothelin-specific CAR NK-92 cells for the treatment of gastric cancer. Int. J. Biol. Sci..

[14] Fitzpatrick E., Filippi C., Jagadisan B., Shivapatham D., Anand H., Lyne M., Stroud K.-D., Newton R., DeLord M., Douiri A. (2023). Intraperitoneal transplant of Hepatocytes co-Encapsulated with mesenchymal stromal cells in modified alginate microbeads for the treatment of acute Liver failure in Pediatric patients (HELP)—An open-label, single-arm Simon’s two stage phase 1 study protocol. PLoS ONE.

[15] Mei J., Sgroi A., Mai G., Baertschiger R., Gonelle-Gispert C., Serre-Beinier V., Morel P., Bühler L.H. (2009). Improved survival of fulminant liver failure by transplantation of microencapsulated cryopreserved porcine hepatocytes in mice. Cell Transplant..

[16] Soon-Shiong P., Heintz R.E., Merideth N., Yao Q.X., Yao Z., Zheng T., Murphy M., Moloney M.K., Schmehl M., Harris M. (1994). Insulin independence in a type 1 diabetic patient after encapsulated islet transplantation. Lancet.

[17] Lim F., Sun A.M. (1980). Microencapsulated islets as bioartificial endocrine pancreas. Science.

[18] González M.A., Gonzalez–Rey E., Rico L., Büscher D., Delgado M. (2009). Adipose-derived mesenchymal stem cells alleviate experimental colitis by inhibiting inflammatory and autoimmune responses. Gastroenterology.

[19] Wang M., Liang C., Hu H., Zhou L., Xu B., Wang X., Han Y., Nie Y., Jia S., Liang J. (2016). Intraperitoneal injection (IP), Intravenous injection (IV) or anal injection (AI)? Best way for mesenchymal stem cells transplantation for colitis. Sci. Rep..

[20] Hunter M.M., Wang A., Parhar K.S., Johnston M.J., Van Rooijen N., Beck P.L., McKay D.M. (2010). In vitro-derived alternatively activated macrophages reduce colonic inflammation in mice. Gastroenterology.

[21] Zani A., Cananzi M., Fascetti-Leon F., Lauriti G., Smith V.V., Bollini S., Ghionzoli M., D’Arrigo A., Pozzobon M., Piccoli M. (2014). Amniotic fluid stem cells improve survival and enhance repair of damaged intestine in necrotising enterocolitis via a COX-2 dependent mechanism. Gut.

[22] Meier R.P., Mahou R., Morel P., Meyer J., Montanari E., Muller Y.D., Christofilopoulos P., Wandrey C., Gonelle-Gispert C., Bühler L.H. (2015). Microencapsulated human mesenchymal stem cells decrease liver fibrosis in mice. J. Hepatol..

[23] Yang C.-Y., Chang P.-Y., Chen J.-Y., Wu B.-S., Yang A.-H., Lee O.K.-S. (2021). Adipose-derived mesenchymal stem cells attenuate dialysis-induced peritoneal fibrosis by modulating macrophage polarization via interleukin-6. Stem Cell Res. Ther..

[24] Wiafe B., Kadam R., Metcalfe P.D. (2020). Intraperitoneal administration of mesenchymal stem cells is effective at mitigating detrusor deterioration after pBOO. Am. J. Physiol. Ren. Physiol..

[25] Flessner M.F. (2005). The transport barrier in intraperitoneal therapy. Am. J. Physiol. Ren. Physiol..

[26] Solass W., Struller F., Horvath P., Königsrainer A., Sipos B., Weinreich F.-J. (2016). Morphology of the peritoneal cavity and pathophysiological consequences. Pleura Peritoneum.

[27] Meza-Perez S., Randall T.D. (2017). Immunological functions of the omentum. Trends Immunol..

[28] Liu M., Silva-Sanchez A., Randall T.D., Meza-Perez S. (2021). Specialized immune responses in the peritoneal cavity and omentum. J. Leucoc. Biol..

[29] Salas-Benito D., Vercher E., Conde E., Glez-Vaz J., Tamayo I., Hervas-Stubbs S. (2020). Inflammation and immunity in ovarian cancer. Eur. J. Cancer Suppl..

[30] Almeida-Nunes D.L., Mendes-Frias A., Silvestre R., Dinis-Oliveira R.J., Ricardo S. (2022). Immune tumor microenvironment in ovarian cancer ascites. Int. J. Mol. Sci..

[31] Mader E.K., Butler G., Dowdy S.C., Mariani A., Knutson K.L., Federspiel M.J., Russell S.J., Galanis E., Dietz A.B., Peng K.-W. (2013). Optimizing patient derived mesenchymal stem cells as virus carriers for a Phase I clinical trial in ovarian cancer. J. Transl. Med..

[32] Olson A., Marini F., Westin S., Coleman R., Thall P., Al Jhadhami V., Qazilbash M.H., Rezvani K., Timmons M., Heese L. (2018). A phase I trial of mesenchymal stem cells transfected with a plasmid secreting interferon Beta in advanced ovarian Cancer. Biol. Blood Marrow Transplant..

[33] Bazhanov N., Ylostalo J.H., Bartosh T.J., Tiblow A., Mohammadipoor A., Foskett A., Prockop D.J. (2016). Intraperitoneally infused human mesenchymal stem cells form aggregates with mouse immune cells and attach to peritoneal organs. Stem Cell Res. Ther..

[34] Preda M.B., Neculachi C.A., Fenyo I.M., Vacaru A.-M., Simionescu M., Burlacu A. (2021). Short lifespan of syngeneic transplanted MSC is a consequence of in vivo apoptosis and immune cell recruitment in mice. Cell Death Dis..

[35] Eich T., Eriksson O., Lundgren T. (2007). Visualization of early engraftment in clinical islet transplantation by positron-emission tomography. N. Engl. J. Med..

[36] Bruni A., Gala-Lopez B., Pepper A.R., Abualhassan N.S., Shapiro A.J. (2014). Islet cell transplantation for the treatment of type 1 diabetes: Recent advances and future challenges. Diabetes Metab. Syndr. Obes. Targets Ther..

[37] Abualhassan N., Sapozhnikov L., Pawlick R.L., Kahana M., Pepper A.R., Bruni A., Gala-Lopez B., Kin T., Mitrani E., Shapiro A.J. (2016). Lung-derived microscaffolds facilitate diabetes reversal after mouse and human intraperitoneal islet transplantation. PLoS ONE.

[38] Gupta S., Rajvanshi P., Sokhi R., Slehria S., Yam A., Kerr A., Novikoff P.M. (1999). Entry and integration of transplanted hepatocytes in rat liver plates occur by disruption of hepatic sinusoidal endothelium. Hepatology.

[39] Lee C.A., Sinha S., Fitzpatrick E., Dhawan A. (2018). Hepatocyte transplantation and advancements in alternative cell sources for liver-based regenerative medicine. J. Mol. Med..

[40] Singh A., Peppas N.A. (2014). Hydrogels and scaffolds for immunomodulation. Adv. Mater..

[41] Roy P., Mignet N., Pocard M., Boudy V. (2021). Drug delivery systems to prevent peritoneal metastasis after surgery of digestives or ovarian carcinoma: A review. Int. J. Pharm..

[42] Tibbitt M.W., Anseth K.S. (2009). Hydrogels as extracellular matrix mimics for 3D cell culture. Biotechnol. Bioeng..

[43] Geckil H., Xu F., Zhang X., Moon S., Demirci U. (2010). Engineering hydrogels as extracellular matrix mimics. Nanomedicine.

[44] Fayzullin A., Bakulina A., Mikaelyan K., Shekhter A., Guller A. (2021). Implantable drug delivery systems and foreign body reaction: Traversing the current clinical landscape. Bioengineering.

[45] Jacobs-Tulleneers-Thevissen D., Chintinne M., Ling Z., Gillard P., Schoonjans L., Delvaux G., Strand B.L., Gorus F., Keymeulen B., Pipeleers D. (2013). Sustained function of alginate-encapsulated human islet cell implants in the peritoneal cavity of mice leading to a pilot study in a type 1 diabetic patient. Diabetologia.

[46] Chen C.-H., Kuo C.-Y., Chen S.-H., Mao S.-H., Chang C.-Y., Shalumon K., Chen J.-P. (2018). Thermosensitive injectable hydrogel for simultaneous intraperitoneal delivery of doxorubicin and prevention of peritoneal adhesion. Int. J. Mol. Sci..

[47] Chen X., Wei Y., Chen X., Zheng L., Zhao Y., You J., Yi C., Yang X. (2026). Hydrogel-Based intraperitoneal drug delivery platforms for peritoneal metastasis: Strategies, advances, and prospects. Drug Deliv..

[48] Annabi N., Tamayol A., Uquillas J.A., Akbari M., Bertassoni L.E., Cha C., Camci-Unal G., Dokmeci M.R., Peppas N.A., Khademhosseini A. (2014). 25th anniversary article: Rational design and applications of hydrogels in regenerative medicine. Adv. Mater..

[49] Wu Z., Su X., Xu Y., Kong B., Sun W., Mi S. (2016). Bioprinting three-dimensional cell-laden tissue constructs with controllable degradation. Sci. Rep..

[50] Ni Y., Qi H., Zhang F., Jiang S., Tang Q., Cai W., Mo W., Miron R.J., Zhang Y. (2023). Macrophages modulate stiffness-related foreign body responses through plasma membrane deformation. Proc. Natl. Acad. Sci. USA.

[51] Zhuang Z., Zhang Y., Sun S., Li Q., Chen K., An C., Wang L., van den Beucken J.J., Wang H. (2020). Control of matrix stiffness using methacrylate–gelatin hydrogels for a macrophage-mediated inflammatory response. ACS Biomater. Sci. Eng..

[52] Arda K., Ciledag N., Aktas E., Arıbas B.K., Köse K. (2011). Quantitative assessment of normal soft-tissue elasticity using shear-wave ultrasound elastography. Am. J. Roentgenol..

[53] Liu J., Zheng H., Poh P.S.P., Machens H.-G., Schilling A.F. (2015). Hydrogels for Engineering of Perfusable Vascular Networks. Int. J. Mol. Sci..

[54] Akbarzadeh R., Yousefi A.M. (2014). Effects of processing parameters in thermally induced phase separation technique on porous architecture of scaffolds for bone tissue engineering. J. Biomed. Mater. Res. Part B Appl. Biomater..

[55] Zhang Y.S., Haghiashtiani G., Hübscher T., Kelly D.J., Lee J.M., Lutolf M., McAlpine M.C., Yeong W.Y., Zenobi-Wong M., Malda J. (2021). 3D extrusion bioprinting. Nat. Rev. Methods Primers.

[56] McCauley P.J., Fromen C.A., Bayles A.V. (2025). Cell viability in extrusion bioprinting: The impact of process parameters, bioink rheology, and cell mechanics. Rheol. Acta.

[57] Tumbleston J.R., Shirvanyants D., Ermoshkin N., Janusziewicz R., Johnson A.R., Kelly D., Chen K., Pinschmidt R., Rolland J.P., Ermoshkin A. (2015). Continuous liquid interface production of 3D objects. Science.

[58] Dhand A.P., Davidson M.D., Burdick J.A. (2025). Lithography-based 3D printing of hydrogels. Nat. Rev. Bioeng..

[59] Carlberg L., Keku I., Zhang Y., Forbes J., Thang M., Perry J., Hingtgen S. (2025). Characterization of a bioprinted anticancer cell therapy system generated with continuous liquid interface production. Adv. NanoBiomed Res..

[60] Hakim Khalili M., Zhang R., Wilson S., Goel S., Impey S.A., Aria A.I. (2023). Additive manufacturing and physicomechanical characteristics of PEGDA hydrogels: Recent advances and perspective for tissue engineering. Polymers.

[61] Ying G., Jiang N., Yu C., Zhang Y.S. (2018). Three-dimensional bioprinting of gelatin methacryloyl (GelMA). Bio Des. Manuf..

[62] Pepelanova I., Kruppa K., Scheper T., Lavrentieva A. (2018). Gelatin-methacryloyl (GelMA) hydrogels with defined degree of functionalization as a versatile toolkit for 3D cell culture and extrusion bioprinting. Bioengineering.

[63] Choi J.R., Yong K.W., Choi J.Y., Cowie A.C. (2019). Recent advances in photo-crosslinkable hydrogels for biomedical applications. BioTechniques.

[64] Bagheri A., Jin J. (2019). Photopolymerization in 3D printing. ACS Appl. Polym. Mater..

[65] Abid A.R., Marciniak B., Pędziński T., Shahid M. (2017). Photo-stability and photo-sensitizing characterization of selected sunscreens’ ingredients. J. Photochem. Photobiol. A Chem..

[66] Xu H., Casillas J., Krishnamoorthy S., Xu C. (2020). Effects of Irgacure 2959 and lithium phenyl-2, 4, 6-trimethylbenzoylphosphinate on cell viability, physical properties, and microstructure in 3D bioprinting of vascular-like constructs. Biomed. Mater..

[67] Mooney R., Abidi W., Batalla-Covello J., Ngai H.W., Hyde C., Machado D., Abdul-Majid A., Kang Y., Hammad M., Flores L. (2021). Allogeneic human neural stem cells for improved therapeutic delivery to peritoneal ovarian cancer. Stem Cell Res. Ther..

[68] Mooney R., Majid A.A., Batalla-Covello J., Machado D., Liu X., Gonzaga J., Tirughana R., Hammad M., Dellinger T.H., Lesniak M.S. (2019). Enhanced delivery of oncolytic adenovirus by neural stem cells for treatment of metastatic ovarian cancer. Mol. Ther. Oncolytics.

[69] Hammad M., Cornejo Y.R., Batalla-Covello J., Majid A.A., Burke C., Liu Z., Yuan Y.-C., Li M., Dellinger T.H., Lu J. (2020). Neural stem cells improve the delivery of oncolytic chimeric orthopoxvirus in a metastatic ovarian cancer model. Mol. Ther. Oncolytics.

[70] Kass L., Thang M., Zhang Y., DeVane C., Logan J., Tessema A., Perry J., Hingtgen S. (2024). Development of a biocompatible 3D hydrogel scaffold using continuous liquid interface production for the delivery of cell therapies to treat recurrent glioblastoma. Bioeng. Transl. Med..

[71] Jiang W., Yang Y., Mercer-Smith A.R., Valdivia A., Bago J.R., Woodell A.S., Buckley A.A., Marand M.H., Qian L., Anders C.K. (2021). Development of next-generation tumor-homing induced neural stem cells to enhance treatment of metastatic cancers. Sci. Adv..

