# Supplementary material for: High-Resolution 3D Bioprinted Hydrogel Scaffolds Enable Sustained Intraperitoneal Cell Delivery
Source: Molecules. 2026 Jun 4;31(11):1958. doi: 10.3390/molecules31111958 (PMC13257711; doi:10.3390/molecules31111958)
Supplement: Supplementary file 1 [file molecules-31-01958-s001.zip › molecules-4211778-supplementary.pdf]

Supplementary Information for:
High-Resolution 3D Bioprinted Hydrogel Scaffolds Enable Sustained Intraperitoneal Cell Delivery

Yu Zhang[1], Lauren E. Carlberg[1], Cali N. Colliver[2], Alain Valdivia[1], Morrent Thang[1], Caroline A. Stockwell[1], Jillian L. Perry[1,2], Shawn D. Hingtgen[1,3]

[1]Division of Pharmacoengineering and Molecular Pharmaceutics, UNC Eshelman School of Pharmacy, The University of North Carolina at Chapel Hill, Chapel Hill, North Carolina, USA

[2]Department of Chemistry, The University of North Carolina at Chapel Hill, Chapel Hill, NC 27599, USA

[3]Center for Nanotechnology in Drug Delivery, Eshelman School of Pharmacy, The University of North Carolina at Chapel Hill, Chapel Hill, North Carolina, USA

[4]Lineberger Comprehensive Cancer Center, The University of North Carolina at Chapel Hill, Chapel Hill, North Carolina, USA

CORRESPONDING AUTHORS CONTACT INFORMATION
Shawn D. Hingtgen, Ph.D.
Division of Pharmacoengineering and Molecular Pharmaceutics
UNC Eshelman School of Pharmacy
University of North Carolina at Chapel Hill
4212 Marsico Hall, 125 Mason Farm Road, Chapel Hill, NC 27599, USA
Email: hingtgen@email.unc.edu

Jillian L. Perry, Ph.D.
Center for Nanotechnology in Drug Delivery
UNC Eshelman School of Pharmacy
University of North Carolina at Chapel Hill
2110 Marsico Hall, 125 Mason Farm Road, Chapel Hill, NC 27599, USA
Email: perryjl@email.unc.edu

KEYWORDS
Continuous Liquid Interface Printing (CLIP), 3D bioprinting, hydrogel scaffolds, cell delivery, intraperitoneal implant

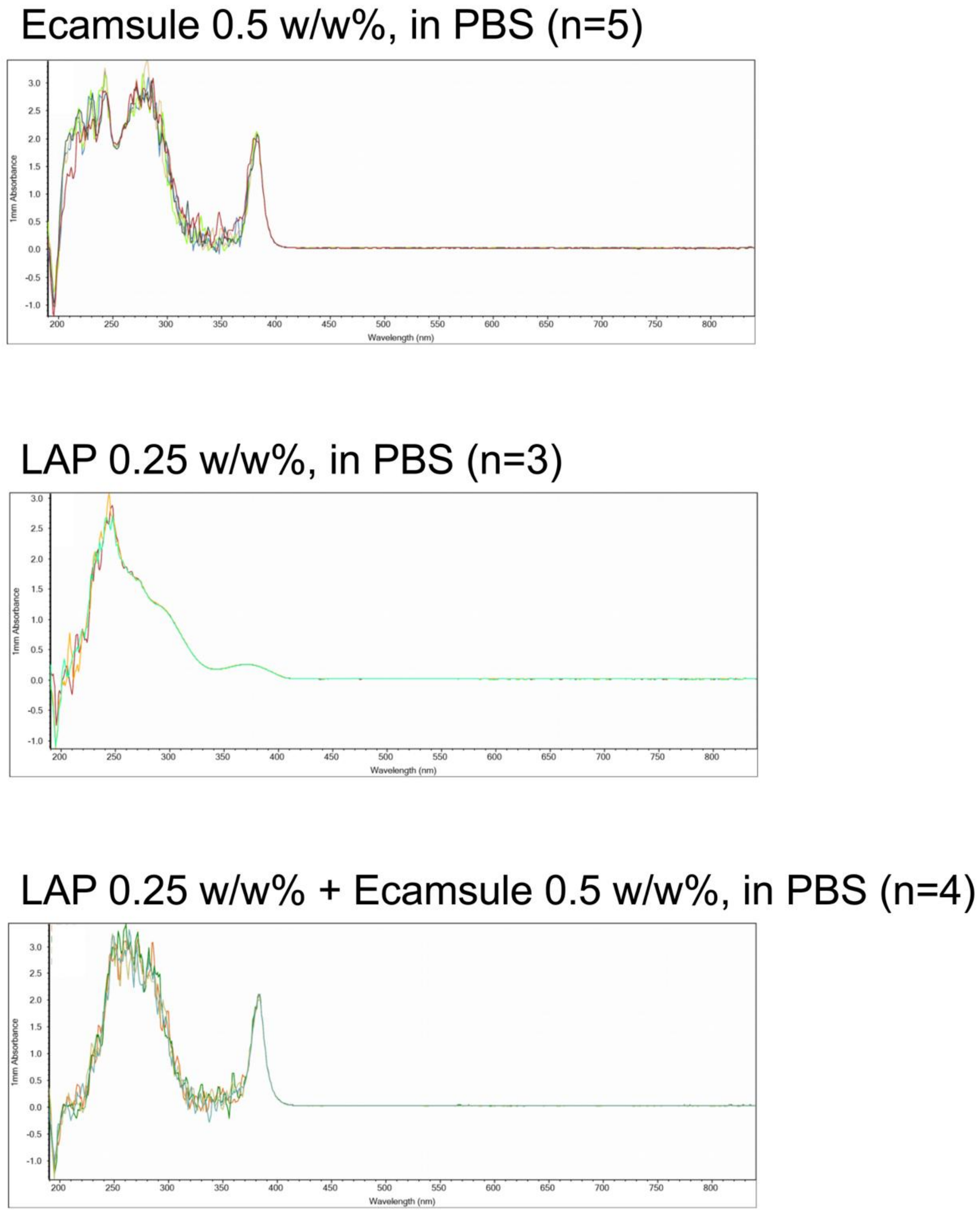

Figure S1 UV-Vis absorption spectra of LAP, Ecamsule, and combined LAP+Ecamsule solutions

UV-Vis absorption spectra of LAP (0.25 w/w%), Ecamsule (0.5 w/w%), and combined LAP/Ecamsule solutions (0.25 w/w% LAP + 0.5 w/w% Ecamsule) prepared in PBS. Solutions were prepared by stirring at 500 rpm at 55 °C until fully dissolved and cooled to room temperature before measurement. UV-Vis absorption spectra were collected using a NanoDrop 2000 spectrophotometer (Thermo Scientific), with PBS used as the blank. For each measurement, 1.2 μL of solution was loaded onto the instrument. Data represent n = 3–5 independent measurements.

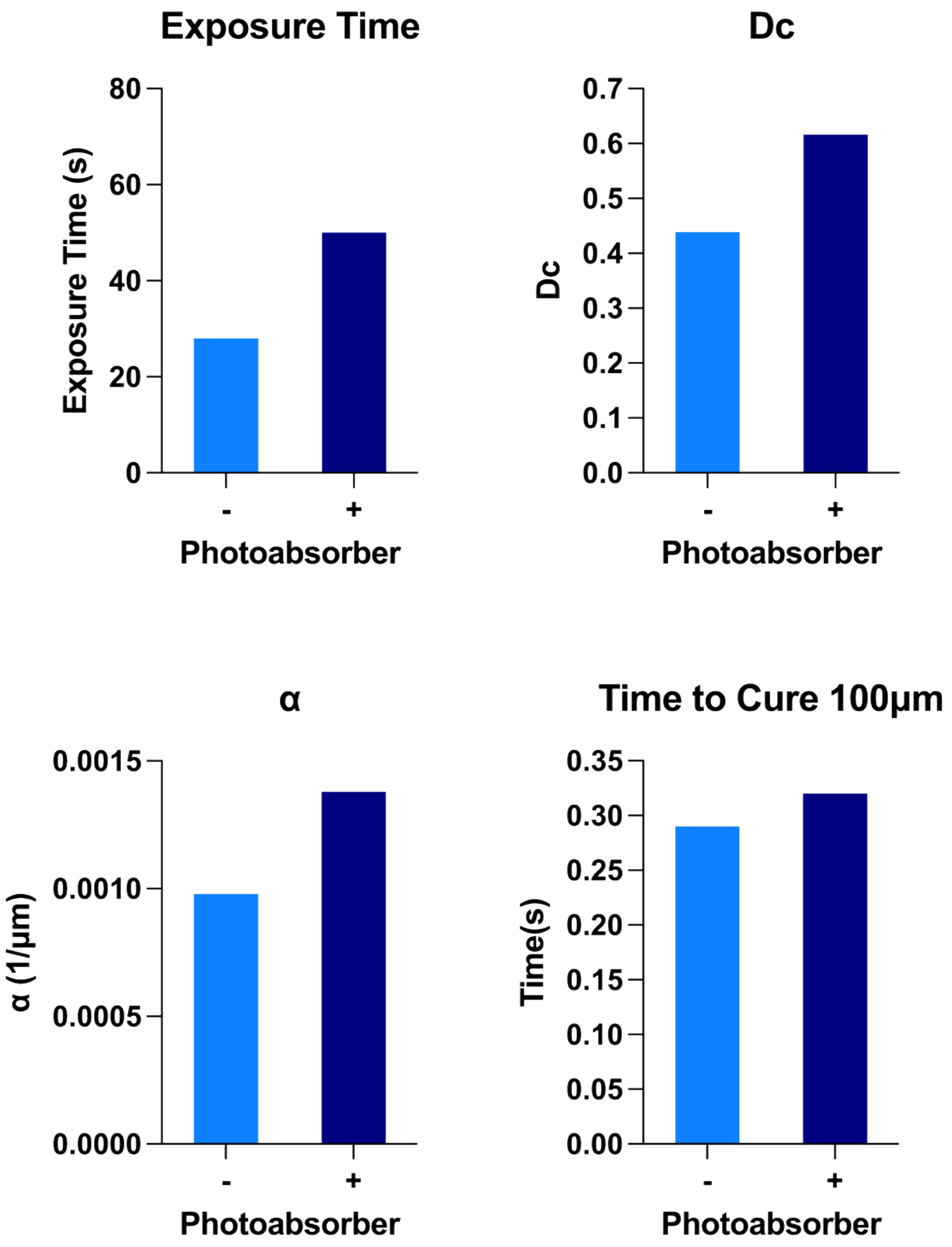

Figure S2 Effect of photoabsorber incorporation on resin curing behavior.
Resin curing behavior was characterized using an in-house curing dosage measurement method, in which a 3×3 array of light intensities was projected through a glass interface onto resin samples to generate cured features under varying exposure conditions. Formulations without photoabsorber (G-P6K) and with photoabsorber incorporation (G-P6K-PA) were evaluated and compared. Cured thickness measurements were analyzed to derive curing-related parameters, including the UV exposure time required for optimal curing assessment, dose-to-cure parameter (Dc), light attenuation parameter (α), and estimated time required to cure a 100 μm feature. Comparison of G-P6K and G-P6K-PA demonstrated that Ecamsule incorporation altered resin curing behavior and effective light attenuation, supporting its role as a photoabsorber in modulating photopolymerization within the CLIP bioresin system.

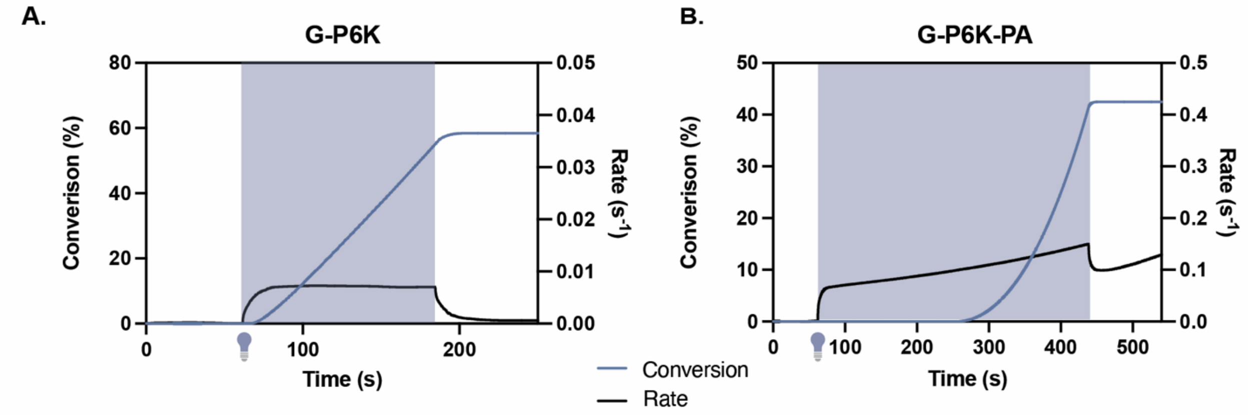

Figure S3 Evaluation of double bond conversion and rate of photocuring via Photo-DSC.

(A) Photocuring rate of G-P6K resin, shown as the rate curve (black), overlaid with calculated double-bond conversion (blue). The shaded region indicates the UV exposure window used to represent a standard printing run for G-P6K (2 min 2 s).

(B) Photocuring rate of G-P6K-PA resin, shown as the rate curve (black), overlaid with calculated double-bond conversion (blue). The shaded region indicates the UV exposure window used to represent a standard printing run for G-P6K-PA (6 min 16 s).

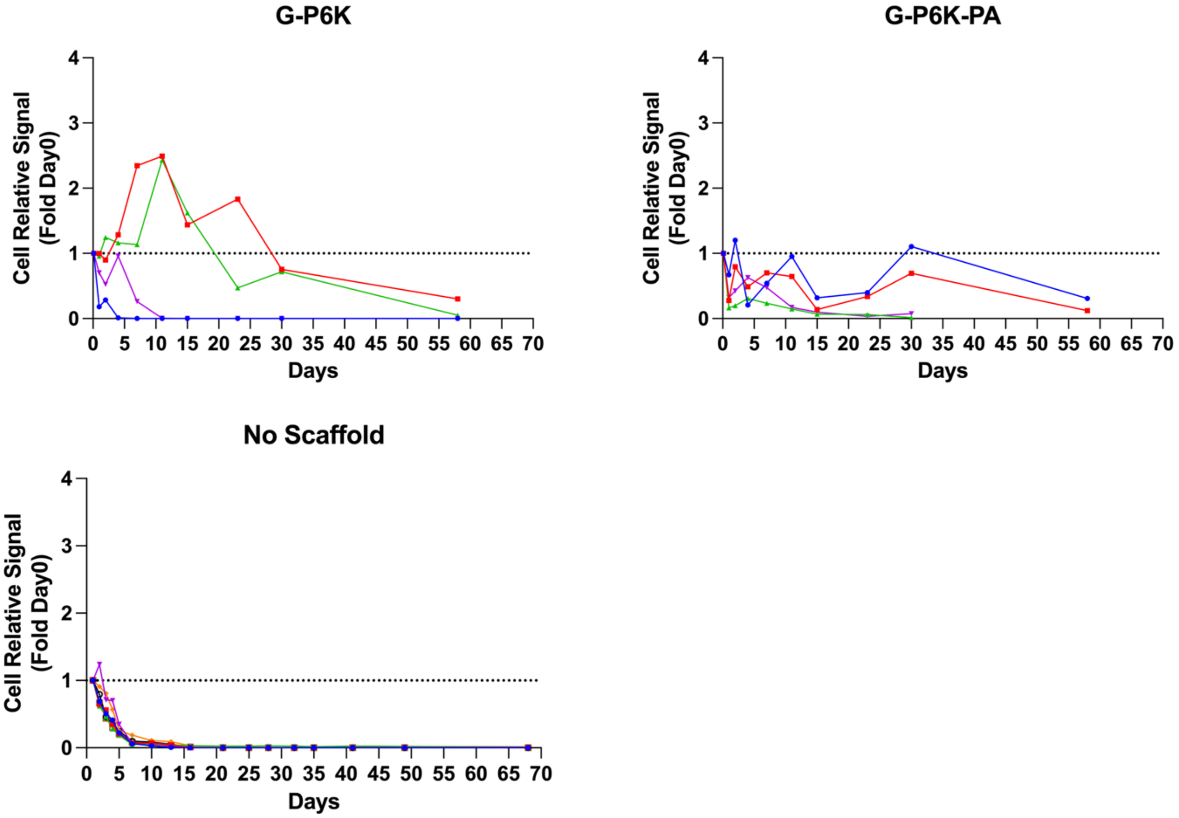

Figure S4 Impact of material composition on *in vivo* cell persistence after intraperitoneal implantation.
Individual BLI signal of NHF1^{GFP-FL} within scaffolds printed with G-P6K-PA and G-P6K in the mouse I.P. cavity, compared with free NHF1^{GFP-FL} injection (No Scaffold). Same data as in Figure 4.

A

Batch	Printing parameters				
	Speed (mm/hr)	Light Intensity (mW/cm²)	Base Delay (s)	Base Exposure (s)	Slice Thickness (um)
1	10	12	10	3	1
2	30	12	10	3	1

B

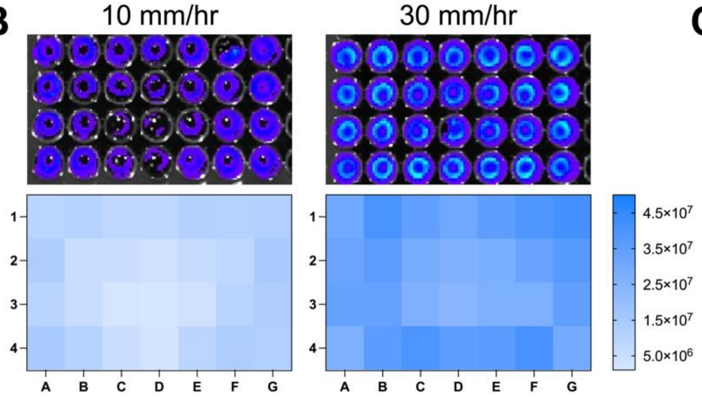

C

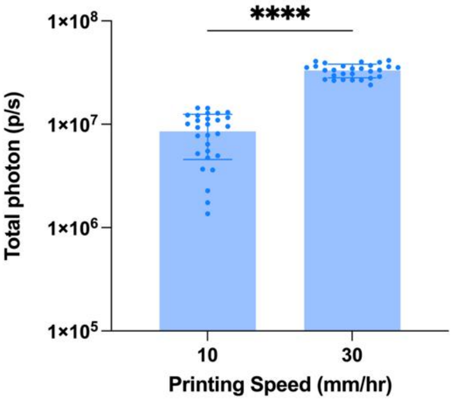

Figure S5 Increasing the printing speed leads to significantly higher cell viability after bioprinting.

$NHF1^{GFP\text{-}FL}$ is bioprinted with G-P6K-PA at a final concentration of 5 x 10^6 cells/mL in 1.5mL bioresin, forming a 4x7 array of scaffolds (Diameter = 5mm, Height = 1mm) on the printing platform.

(A) Table of printing parameters are shown for batch 1 and 2, the main difference being printing speed.

(B) For batch 1 (printing speed 10 mm/hr) and batch 2 (printing speed 30mm/hr), IVIS image of all scaffolds within one batch of printing (top) and BLI signal distribution in a heat map (bottom) is shown.

(C) BLI signals of NHF1 in scaffolds from each batch are compared.

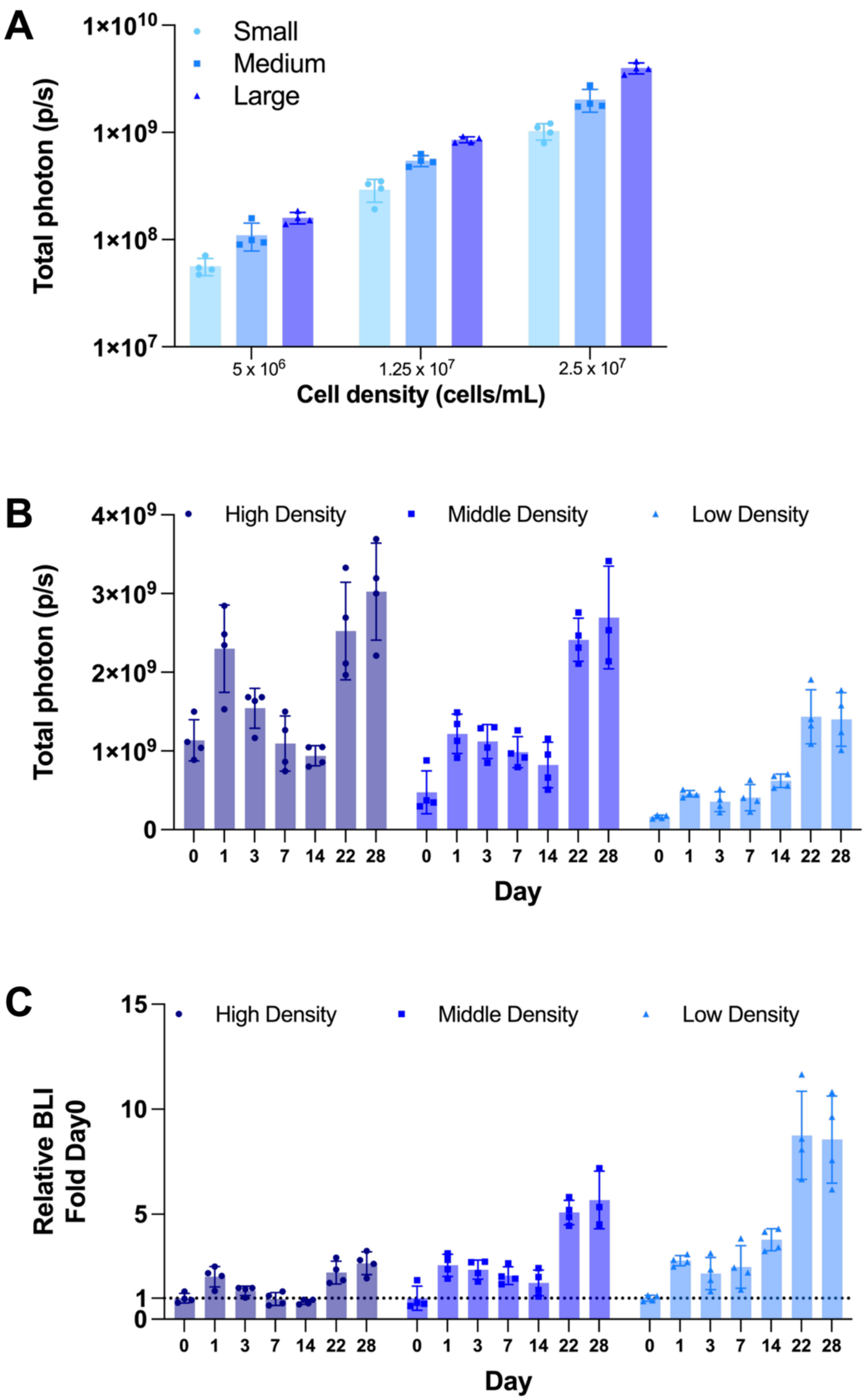

Figure S6 Tuning cell loading in CLIP 3D-bioprinted scaffolds.
(A) Absolute individual BLI signal of NHF1s bioprinted with G-P6K-PA and different scaffold sizes. Same data as in Figure 6A. Absolute (B) and relative (C) longitudinal BLI signal of NHF1s bioprinted with G-P6K-PA at various cell densities. Same data as in Figure 6C and 6D.

Table S1 Summary of resin formulation variables and key performance outcomes

	G	P6K	P10K	G-P6K	G-P6K-PA
GelMA (w/w%)	5			2.5	2.5
PEGDA6K (w/w%)		10		5	5
PEGDA10K (w/w%)			10		
Ecamsule					0.5
Printing Resolution	-	+	+	++	+++
Cell Encapsulation & Proliferation	+++	-	-	+++	+++
In vivo cell persistence	NA	NA	NA	+++	+++
